# Structural and Functional Analysis of Murine Polyomavirus Capsid Proteins Establish the Determinants of Ligand Recognition and Pathogenicity

**DOI:** 10.1371/journal.ppat.1005104

**Published:** 2015-10-16

**Authors:** Michael H. C. Buch, A. Manuel Liaci, Samantha D. O’Hara, Robert L. Garcea, Ursula Neu, Thilo Stehle

**Affiliations:** 1 Interfaculty Institute of Biochemistry, University of Tübingen, Tübingen, Germany; 2 Department of Molecular, Cellular, and Developmental Biology, and the BioFrontiers Institute, University of Colorado, Boulder, Colorado, United States of America; 3 Department of Pediatrics, Vanderbilt University School of Medicine, Nashville, Tennessee, United States of America; Penn State University School of Medicine, UNITED STATES

## Abstract

Murine polyomavirus (MuPyV) causes tumors of various origins in newborn mice and hamsters. Infection is initiated by attachment of the virus to ganglioside receptors at the cell surface. Single amino acid exchanges in the receptor-binding pocket of the major capsid protein VP1 are known to drastically alter tumorigenicity and spread in closely related MuPyV strains. The virus represents a rare example of differential receptor recognition directly influencing viral pathogenicity, although the factors underlying these differences remain unclear. We performed structural and functional analyses of three MuPyV strains with strikingly different pathogenicities: the low-tumorigenicity strain RA, the high-pathogenicity strain PTA, and the rapidly growing, lethal laboratory isolate strain LID. Using ganglioside deficient mouse embryo fibroblasts, we show that addition of specific gangliosides restores infectability for all strains, and we uncover a complex relationship between virus attachment and infection. We identify a new infectious ganglioside receptor that carries an additional linear [α-2,8]-linked sialic acid. Crystal structures of all three strains complexed with representative oligosaccharides from the three main pathways of ganglioside biosynthesis provide the molecular basis of receptor recognition. All strains bind to a range of sialylated glycans featuring the central [α-2,3]-linked sialic acid present in the established receptors GD1a and GT1b, but the presence of additional sialic acids modulates binding. An extra [α-2,8]-linked sialic acid engages a protein pocket that is conserved among the three strains, while another, [α-2,6]-linked branching sialic acid lies near the strain-defining amino acids but can be accommodated by all strains. By comparing electron density of the oligosaccharides within the binding pockets at various concentrations, we show that the [α-2,8]-linked sialic acid increases the strength of binding. Moreover, the amino acid exchanges have subtle effects on their affinity for the validated receptor GD1a. Our results indicate that both receptor specificity and affinity influence MuPyV pathogenesis.

## Author Summary

Viruses are obligate intracellular pathogens, and all of them share one crucial step in their life cycle—the attachment to their host cell via cellular receptors, which are usually proteins or carbohydrates. This step is decisive for the selection of target cells and virus entry. In this study, we investigated murine polyomavirus (MuPyV), which attaches to host gangliosides with its major capsid protein, VP1. We have solved the crystal structures of VP1 in complex with previously known interaction partners as well as with the ganglioside GT1a, which we have identified as a novel functional receptor for MuPyV. Earlier studies have shown that different strains with singular amino acid exchanges in the receptor binding pocket of VP1 display altered pathogenicity and viral spread. Our investigations show that, while these exchanges do not abolish binding or significantly alter interaction modes to our investigated carbohydrates, they have subtle effects on glycan affinity. The combination of receptor specificity, abundance, and affinity reveals a much more intricate regulation of pathogenicity than previously believed. Our results exemplify how delicate changes to the receptor binding pocket of MuPyV VP1 are able to drastically alter virus behavior. This system provides a unique example to study how the first step in the life cycle of a virus can dictate its biological properties.

## Introduction

**Fig 1:**
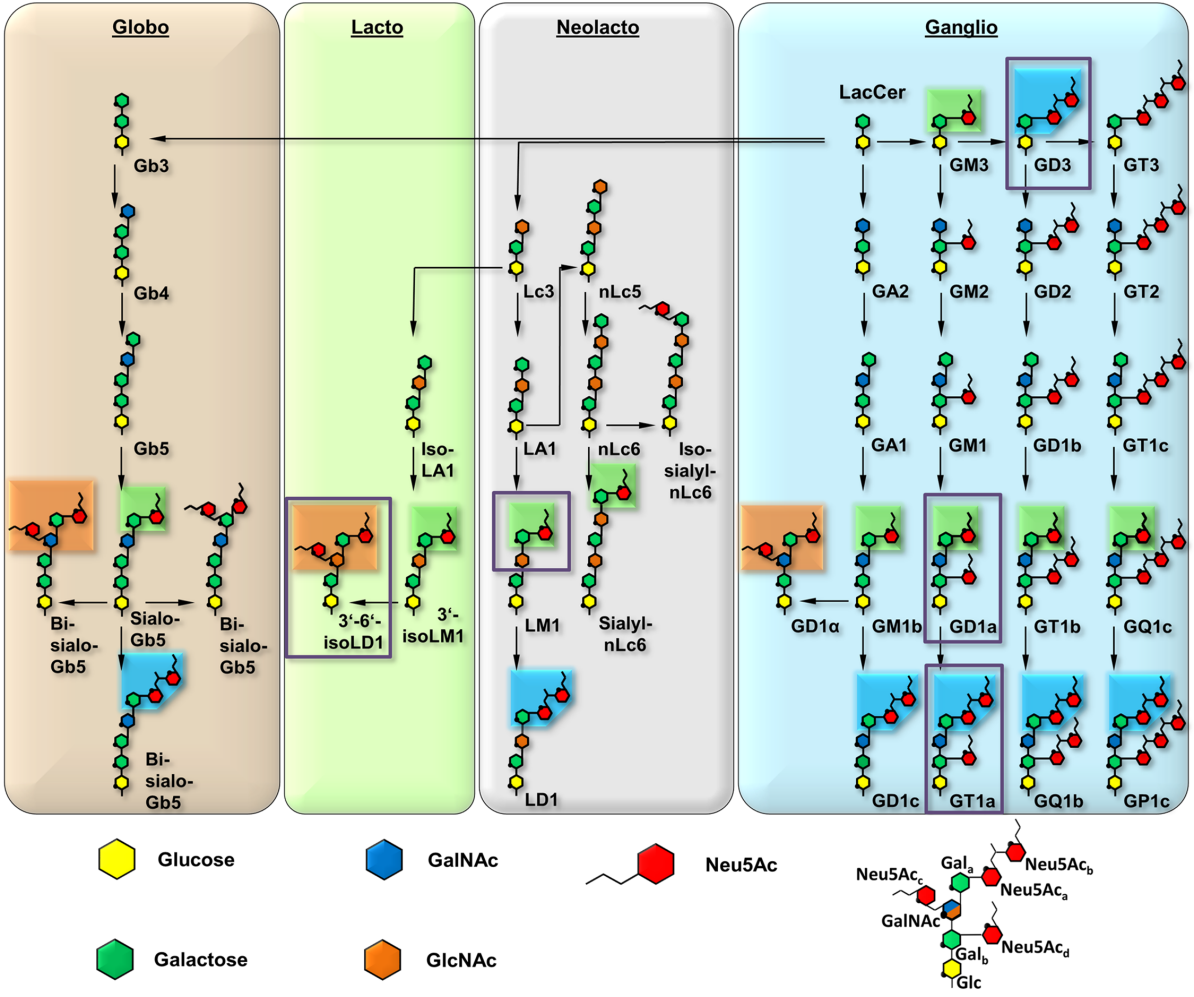
Overview and biosynthetic pathway of the four most prominent ganglioside series. The glycan parts of important members are shown for each series. The downstream biosynthetic steps are identical for all members of a row, although they may vary in linkage orientation. The six-membered pyranose rings are numbered counterclockwise, starting from the bottom (C1, except for C2 in Neu5Ac), and the ring oxygen is symbolized with a black dot. Neu5Ac moieties are rearranged for clarity, and all linkages are mediated by O2 or O8. Most of the gangliosides (e.g. LM1) can be further modified, e.g. by fucosylation. Linkages involving Neu5Ac are present in the α conformation, all other linkages are in the β conformation. Boxes represent three distinguishable sialoglycotopes that contain linkages found in GT1a (blue, representative for [α-2,8]), GD1a (green, [α-2,3]), and 3’-6’-iso-LD1 (also referred to as DSLNT, orange, [α-2,6]). The naming is according to the corresponding gangliosides; if possible, the Svennerholm shorthand is used [64–66] All biosynthetic routes were verified using the KEGG metabolic pathway database [67]. A prototype glycan that exemplifies the different positions of Gal and Neu5Ac moieties is depicted on the lower right. The glycan portions investigated in this study are highlighted by purple boxes.

The engagement of one or several host cell receptors is the first step in the infectious cycle of a virus. A large number of viruses, including many human pathogens, depend on carbohydrate recognition for initial attachment to the cell surface. Viral tropism and the internalization pathway are usually determined by the specificity and affinity of the receptor interaction as well as the glycan distribution on different cell surfaces (reviewed in [1]). Many viruses use glycoproteins, glycolipids, or both as receptors for cell entry [2]. Gangliosides are ubiquitous glycolipids on the outer leaflet of mammalian cell membranes that serve as receptors for a number of viruses. They are composed of a membrane-embedded ceramide moiety linked to a complex carbohydrate structure that projects away from the cell. Gangliosides almost always contain α-5-*N*-acetyl-neuraminic acid (sialic acid, Neu5Ac) that can be attached to the core of the molecule with [α-2,3], [α-2,6], or [α-2,8] linkages (Fig 1). Gangliosides exist on cell surfaces in complex and poorly understood patterns that are cell type-, age-, and tissue-dependent ([3,4], reviewed in [5]).

Murine Polyomavirus (MuPyV) is a double-stranded DNA virus that can induce tumors in newborn animals. It was long known to engage glycan receptors that contain a minimal motif of sialic acid [α-2,3]-linked to galactose [6,7], and more recently gangliosides GD1a and GT1b were identified as MuPyV receptors [8]. Viral attachment is mediated by the major capsid protein, VP1, which forms pentameric capsomers that assemble into the T = 7*d* icosahedral capsid of the virus [9,10]. Sialylated oligosaccharide receptors are engaged in a shallow groove on top of VP1 formed by loop structures on the protein surface [11–13], similar to other polyomaviruses [1].

MuPyV displays striking differences in pathogenicity and spread among three closely related prototype strains upon infection of newborn virus-free mice. The laboratory-derived RA strain [14] shows limited spread and induces few tumors of strictly mesenchymal origin after a long latency period, while the naturally occurring PTA strain [15,16] has disseminated infection and causes multiple tumors of epithelial and mesenchymal origin within a short time. LID [17,18], another laboratory isolate MuPyV strain, spreads most rapidly, causing early death by damaging host tissues, leading to brain hemorrhages and kidney failure [19]. The differences among the three strains have been mapped to amino acid variations at two positions, 91 and 296, within the receptor-binding region of VP1 [20–24]. While RA bears a glycine residue at position 91, this residue is replaced with a glutamate in both PTA and LID. An additional valine-to-alanine exchange at position 296 is present in LID (Table 1). The pathogenicity profile of one strain can be introduced into the other strains by mutating these two residues, confirming that these substitutions are necessary and sufficient to generate a specific phenotype [25]. The same substitutions have also been observed for other strains of MuPyV [21,22]. MuPyV found in feral mice has the VP1 sequence of PTA [26], but the virus is controlled by an intact immune system. As studies of viral spread can be conducted *in vivo* and virus infectivity can be tested *in vitro* using ganglioside deficient mouse cells, MuPyV represents an attractive and rare model system to define the relationships between receptor binding and viral spread and tropism.

**Table 1:** Description of the investigated MuPyV strains.

	RA Strain	PTA Strain	LID Strain
**Distinctive amino acids**	G91, V296	E91, V296	E91, A296
**Pathogenicity**	No or only singular tumors, mesenchymal origin.	High tumor density of epithelial and mesenchymal origin.	Virulent. Damage of host tissues, early death due to brain hemorrhages and kidney failure.
**Latency**	Long	Short	Very short

Crystal structures of the low pathogenicity strain RA have shown how this virus engages 3’-sialyllactose, a short, linear trisaccharide terminating in [α-2,3]-linked sialic acid, as well as an oligosaccharide that additionally contains a second, branching [α-2,6]-linked sialic acid [11,12]. These structures also identified the location of residues 91 and 296 in the carbohydrate-binding region, suggesting that they might modulate interactions of VP1 with its receptors in the higher pathogenicity strains PTA and LID. Modelling suggested that a glutamate side chain at position 91 would lead to electrostatic repulsion of the [α-2,6]-branched sialic acid, thereby preventing binding of such branched structures by either LID or PTA. Branched sugars carrying an [α-2,6]-linked sialic acid could thus act as pseudoreceptors that will not facilitate productive infection but hamper the spread of RA within the host, in contrast to PTA and LID [8,12]. In line with this hypothesis, gangliosides GD1a and GT1b, which do not contain an [α-2,6]-branched sialic acid, have been identified as entry receptors for the PTA [8,16] and RA strain [27] of MuPyV. However, the molecular determinants of GD1a or GT1b receptor interactions with PTA and LID are not understood, because all structural information is limited to date to RA MuPyV.

To define the interactions of the three MuPyV strains with receptors on the cell surface, we have solved high-resolution structures of RA, PTA, and LID VP1 pentamers in complex with three ganglioside glycans that represent common motifs found in members of the four most prominent ganglioside biosynthesis series and that feature [α-2,3]-, [α-2,6]-, and [α-2,8]-linked sialic acids (for carbohydrate structures, nomenclature, and annotations see Fig 1). We have also conducted crystallographic soaking experiments at different ligand concentrations to compare the relative affinities of each of the three strains for their interaction partners. We find that expanding the well-characterized Neu5Ac-[α-2,3]-Gal epitope with a linear [α-2,8]-linked sialic acid (as found for example in GT1a vs. GD1a) leads to additional interactions between carbohydrate and VP1 in all three strains. Consequently, we identify ganglioside GT1a as an infectious receptor for all three strains. Moreover, the branching [α-2,6]-linked sialic acid is close to the strain-defining amino acids, but can be accommodated by all strains, in contrast to the earlier model. However, the amino acid exchanges defining each strain have subtle effects on their affinity for the validated receptor GD1a. Our results exemplify the effect of minimal changes in a binding pocket on the receptor binding properties of a virus.

## Results

### GT1a, GD1a, and GT1b gangliosides are infectious receptors for MuPyV

**Fig 2:**
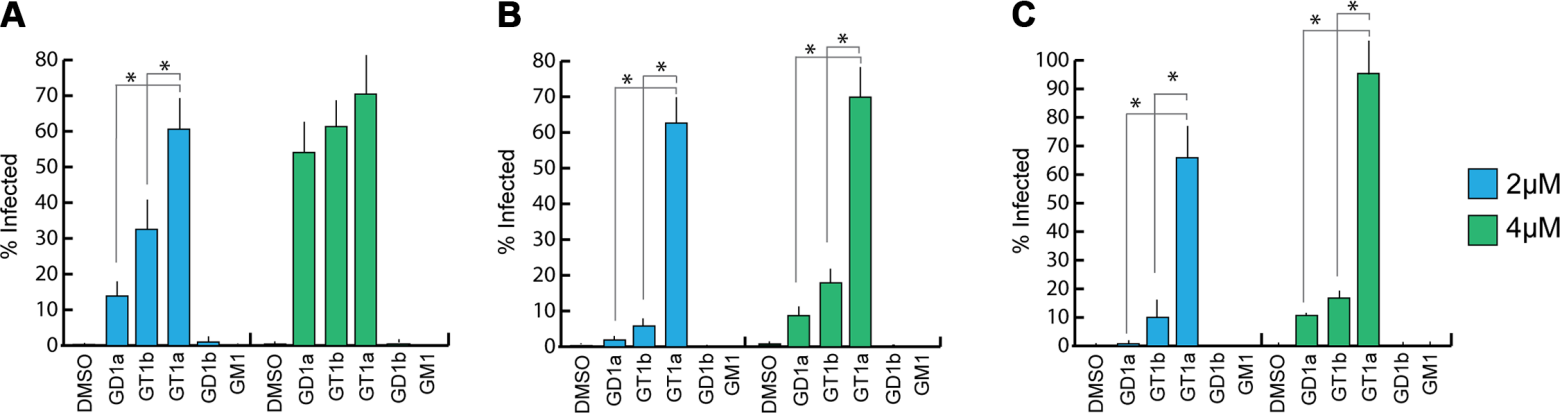
GT1a, GT1b, and GD1a supplementation rescues MuPyV infection of Gang-/- MEFs. Ganglioside knock-out (Gang-/-) MEFs were completely resistant to infection of all strains of MuPyV as shown by the absence of T-antigen positive nuclei at 24 hours post infection (DMSO control). GD1a, GT1b, and GT1a ganglioside supplementation of Gang-/- MEFs restored RA (**A**), PTA (**B**), and LID MuPyV (**C**) infection, while GD1b and GM1 supplementation resulted in little to no infection by any virus strain. Infection levels were quantified at both 2 μM and 4 μM ganglioside supplementation (blue and green bars, respectively). Infection levels are normalized to MuPyV infection of WT MEFs, and error bars correspond to standard error.

Previous efforts to identify receptors for MuPyV used immortalized cell lines, such as Vero or C6 glioma cells that were supplemented with candidate gangliosides before infection [8,28]. We utilized a mouse embryo knock-out fibroblast cell line (Gang-/- MEFs) specifically deficient in ganglioside synthesis and completely resistant to MuPyV infection (S1A Fig and [29]) to test the ability of ganglioside receptors to rescue infection by different strains of MuPyV. Gang-/- MEFs were supplemented with individual gangliosides followed by infection with RA, PTA, and LID MuPyV (Fig 2). Importantly, it should be noted that the three MuPyV strains we used do not have the same particle to PFU ratio. The viruses have been normalized to similar MOIs, but they cannot be quantitatively compared to one another. However, each strain has been normalized to its own infection rate of WT MEFs; therefore, infection rates upon supplementation of gangliosides can be compared within a strain. The previously identified ganglioside receptors GD1a and GT1b [8] rescued RA, PTA, and LID infection of Gang-/- MEFs in a dose-responsive manner. We also analyzed the GT1a ganglioside that had not been previously investigated as a candidate infectious receptor for MuPyV. We found that GT1a, a member of the ganglio-series synthesized from GD1a (Fig 1), also rescued RA, PTA, and LID infection in a dose responsive manner (Fig 2). Moreover, GT1a supplementation of Gang-/- MEFs conferred higher levels of RA, PTA, and LID MuPyV infection than the previously identified receptors GD1a and GT1b. Finally, we tested the ability of the gangliosides GD1b and GM1 to rescue MuPyV infection of Gang-/- MEFs. GD1b and GT1b supplementation has previously been shown to restore BK polyomavirus infection of ganglioside deficient cells [30]; however, GD1b restored little to no MuPyV infection of Gang-/- MEFs. GM1 supplementation has previously been shown to restore infection by SV40 [8]; however, GM1 did not rescue MuPyV infection of Gang-/- MEFs. These data confirm that GT1a is an infectious receptor for all strains of MuPyV.

We also investigated whether MuPyV cell surface binding to infectious or non-infectious ganglioside receptors correlated with infection. To this end, we measured the levels of free (unbound) virus in each ganglioside supplemented sample at 4 hours post infection. We did not detect significant differences in MuPyV cell surface binding to different ganglioside receptors or WT MEFs, indicating that cell surface binding alone does not determine infection (S1B Fig). Instead, a considerable amount of virus binds to Gang-/- MEFs even in the absence of ganglioside supplementation (S1A Fig). MuPyV is also endocytosed in Gang-/- MEFs, which however does not lead to infection [29]. Taken together, these data confirm that gangliosides are not required for cell surface binding. They are, however, required for infection, and GT1a appears to be more efficient than GD1a and GT1b.

### Structure of MuPyV VP1 bound to GT1a

**Fig 3:**
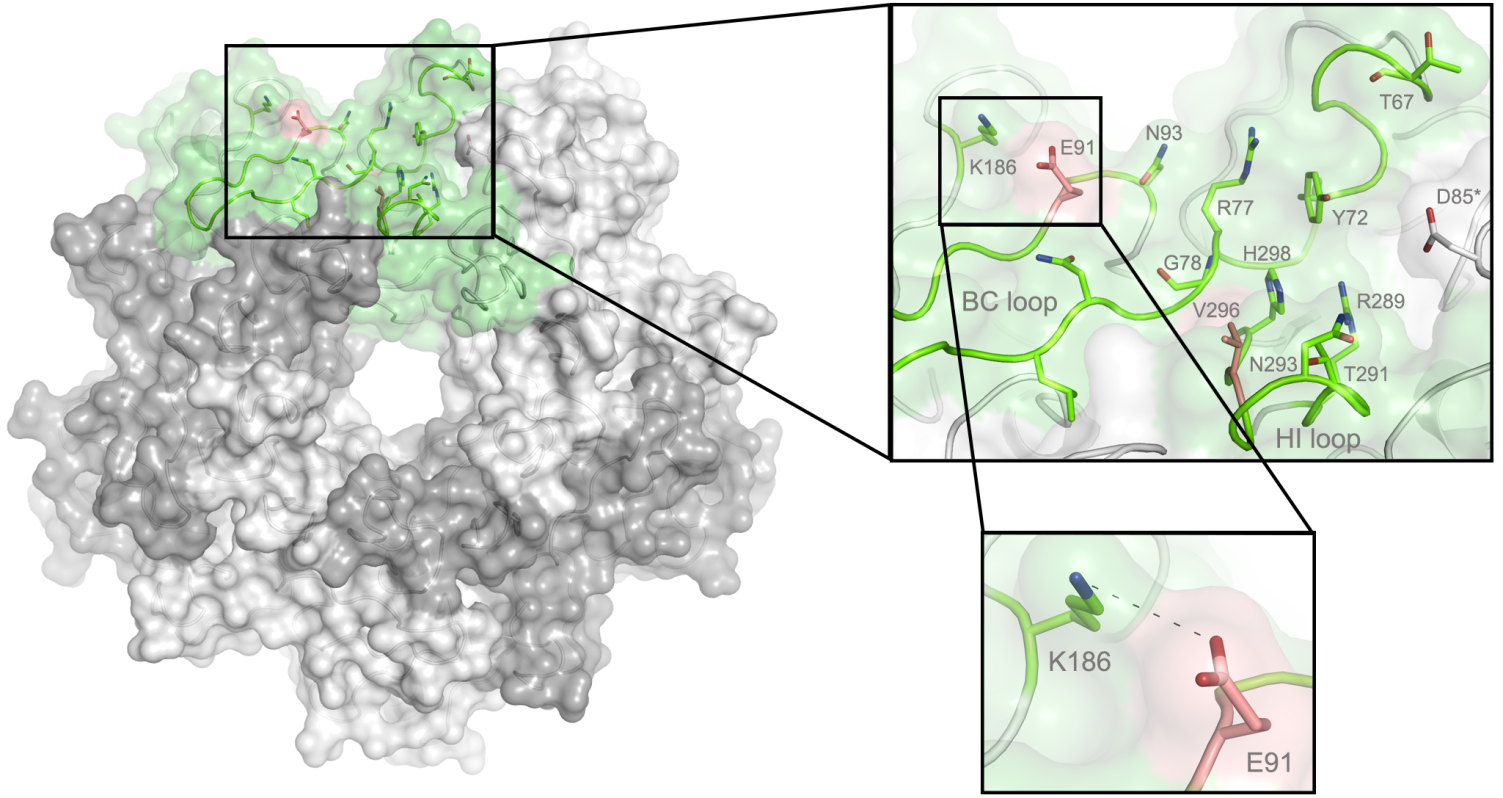
The MuPyV binding pocket. Top view on the receptor-binding region of PTA, which is shown with E91 and V296 highlighted in salmon. Residues that are known to participate in receptor binding are contributed by the BC and HI loops and are highlighted as stick models. One monomer is shaded in green and the other monomers are alternatingly shaded light and dark grey for better distinction.

**Fig 4:**
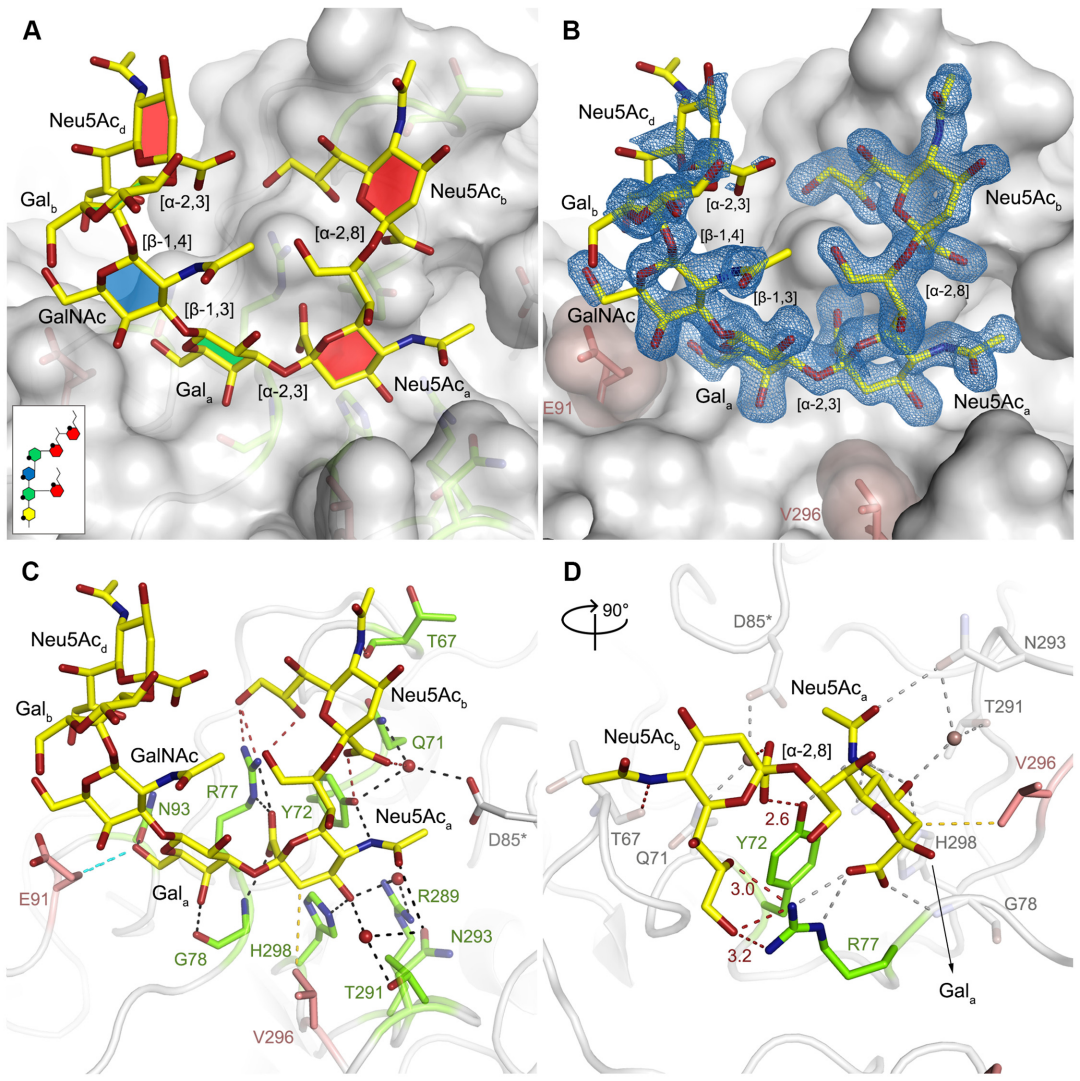
Binding of GT1a to PTA. **A** The PTA binding pocket and the GT1a conformation upon binding are shown from an angle parallel to the fivefold axis. A scheme of the glycan is shown in the inset, and the sugar rings are filled according to the coloring scheme from Fig 1. **B** Simulated annealing F_obs_-F_calc_ omit map (resolution 1.71 Å, calculated at 3.5 σ, carved 2.3 Å around the glycan). **C** Possible binding interactions of GT1a and PTA. E91 and V296 are highlighted in salmon. Hydrogen bonds are shown in black, the hydrophobic contact mediated by V296 in gold, and the van-der-Waals contacts of E91 are shown in cyan. Waters that mediate key hydrogen bonds are shown as red spheres. Unique interactions mediated by the novel GT1a-like binding motif are shown in red. **D** Zoomed view of the binding to the two terminal Neu5Ac moieties. The rest of the glycan is omitted for clarity. Residues except Y72 and R77 as well as waters involved in contacts with these two glycan moieties are pale grey and salmon, respectively.

In order to define the mode of recognition of GT1a, particularly to the naturally occurring PTA strain of MuPyV, we have soaked VP1 crystals with the glycan portion of GT1a and solved the structure of the complex (Table 2). While the receptor interaction pocket of RA VP1 has been described [11–13], no structural information for the pathogenicity-defining amino acids at positions 91 and 296 in the pockets of PTA and LID has been available. PTA and LID both carry a glutamate at position 91, and this side chain is being held in a characteristic position with the carboxyl group facing away from the glycan receptor due to a salt bridge formed with K186 (Fig 3), as previously predicted [12]. The GT1a glycan is a branched structure with a long disialylated arm, which has the sequence Neu5Ac_b_-[α-2,8]-Neu5Ac_a_-[α-2,3]-Gal_a_-[β-1,3]-GalNAc, and a second short arm, which consists of a single Neu5Ac_d_ [α-2,3]-linked to Gal_b_ (for carbohydrate structures, nomenclature, and moiety indexing see Fig 1). The disialylated arm of GT1a is clearly visible in the crystal structure of PTA VP1; it is well defined by electron density and makes extensive contacts with the protein (Fig 4B–4D). Overall, the GT1a glycan adopts a twisted horseshoe-like shape, with Neu5Ac_a_ and Neu5Ac_b_ wrapping around the side chains of Y72 and R77 of VP1. Its longer, disialylated arm contains a Neu5Ac_a_-[α-2,3]-Gal_a_ sequence that is also present in GD1a and simpler compounds such as 3’-sialyllactose (3SL), and the interactions of this motif with VP1 are essentially identical to those seen in previous structures [11–13]. However, our structure visualizes an additional network of contacts made by the terminal [α-2,8]-linked Neu5Ac_b_ (Fig 4C and 4D). Its carboxyl group engages Y72 and forms water-mediated hydrogen bonds with Q71, Y72, as well as D85 of the neighboring monomer (D85*). In addition, the *N*-acetyl nitrogen of Neu5Ac_b_ forms a hydrogen bond with the backbone carbonyl of T67, and O8 and O9 in the glycerol chain of the sugar are hydrogen-bonded with the R77 side chain. The carboxyl groups of Neu5Ac_a_ and Neu5Ac_b_ are about 4 Å apart, and the positively charged side chain of R77 counteracts their negative charges (Fig 4C and 4D). Neu5Ac_a_ and Neu5Ac_b_ contribute binding interfaces of approximately 160 Å^2^ and 190 Å^2^, respectively (calculated using the PISA server [31]). The remaining Gal_a_-GalNAc-Gal_b_ stem of GT1a forms fewer contacts with the protein, which include a hydrogen bond between G78 and the Gal_a_ O4 hydroxyl group (Fig 4) as well as several van der Waals interactions. Notably, the C_β_ and C_γ_ atoms of E91 are within van-der-Waals range of O6 and C6 of Gal_a_, and the E91 carboxylate group is close to C6 of GalNAc. The total contact surface for this portion of the glycan is 142 Å^2^.

**Table 2:** Data collection and refinement statistics.

	PTA VP1 Native	PTA VP1 + GT1a	PTA VP1 + GD1a	PTA VP1 + DSLNT	RA VP1 + GT1a	RA VP1 + GD1a
**Data Collection**						
Beamline	SLS, X06DA	SLS, X06DA	SLS, X06DA	ESRF, ID 14–1	SLS, X06DA	SLS, X06DA
Space Group	P3_1_21	P3_1_21	P3_1_21	P3_1_21	P3_1_21	P3_1_21
Cell Dimensions						
a = b, c [Å]	219.61, 99.82	219.60, 99.74	220.45, 99.71	219.73, 100.00	219.55, 99.60	219.06, 99.40
α = β, γ [°]	90, 120	90, 120	90, 120	90, 120	90, 120	90, 120
Resolution [Å]	50–1.64 (1.68–1.64)	50–1.75 (1.79–1.75)	50–1.93 (1.98–1.93)	50–1.87 (1.92–1.87)	50–1.71 (1.75–1.71)	50–1.90 (1.95–1.90)
R_meas_ [%]	10.5 (68.7)	13.3 (68.3)	11.3 (68.8)	15.2 (69.1)	7.4 (74.1)	11.1 (71.7)
I/σ(I)	10.5 (2.3)	7.08 (1.51)	12.8 (3.0)	7.6 (3.0)	15.71 (2.17)	11.3 (1.9)
Completeness [%]	99.9 (99.9)	97.6 (96.6)	96.0 (98.1)	99.8 (99.8)	99.8 (99.9)	99.0 (99.1)
Redundancy	5.0 (4.8)	3.4 (3.2)	5.4 (5.5)	3.7 (3.7)	4.3 (3.9)	2.9 (2.8)
Wilson B-Factor [Å^2^]	23.1	25.3	25.7	23.1	26.0	23.3
**Refinement**						
Resolution [Å]	48.2–1.64	47.6–1.75	47.8–1.93	48.4–1.83	50–1.71	48.1–1.90
No. of Reflections	324,802	261,253	192,327	220,105	285,887	205,733
R_work_ / R_free_ [%]	15.85 / 17.30	16.0 / 18.13	15.27 / 17.42	15.38 / 17.56	15.27 / 16.98	15.46 / 17.84
No. of Atoms						
Protein	11,117	11,088	11,150	10,996	11,323	11,225
Solvent	1,840	1,827	1,884	1,632	2,059	1,971
Carbohydrate	-	425	285	202	385	285
B-Factors [Å^2^]						
Protein	19.3	20.7	21.4	20.1	21.1	19.9
Solvent	29.6	30.9	31.5	30.5	32.6	31.1
Carbohydrate	-	30.3	35.1	34.2	36.0	38.2
R. m. s. Deviations				** **		
Bond Lengths [Å]	0.007	0.007	0.006	0.008	0.007	0.008
Bond Angles [°]	1.16	1.17	1.06	1.20	1.13	1.19
Ramachandran Plot				** **		
Favored	1,340 (97.2%)	1,336 (97.0%)	1,335 (96.9%)	1,334 (96.9%)	1,342 (97.0%)	1,340 (97.0%)
Allowed	38 (2.8%)	41 (3.0%)	42 (3.2%)	42 (3.1%)	42 (3.0%)	42 (3.0%)
Disallowed	0 (0%)	0 (0%)	0 (0%)	0 (0%)	0 (0%)	0 (0%)
PDB ID	5CPU	5CPW	5CPY	5CPX	5CPZ	5CQ0

**Fig 5:**
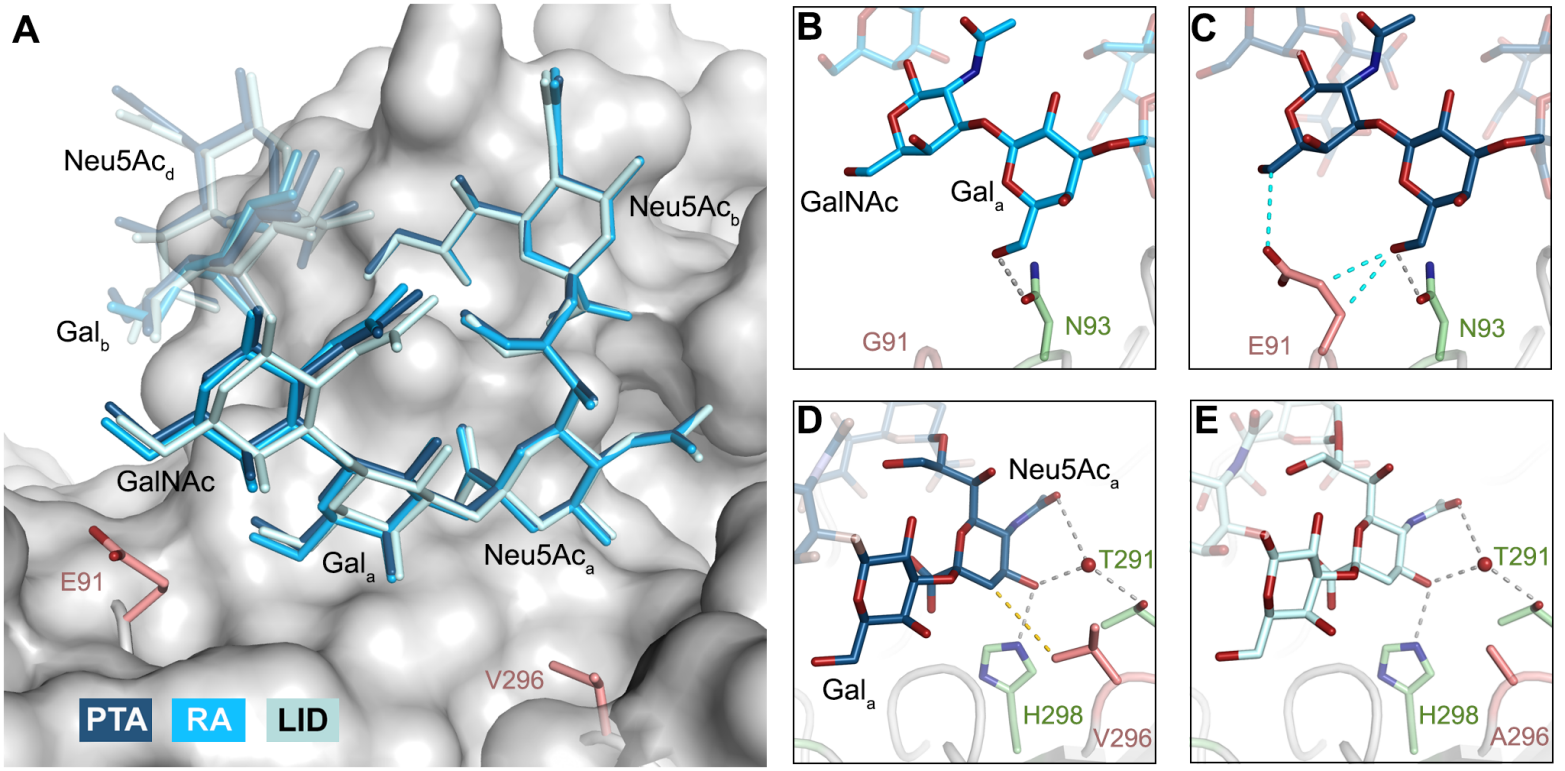
Binding modes of GT1a to the different MuPyV strains. **A** Superposition of the GT1a-binding mode of RA (GT1a in sky blue), PTA (dark blue), and LID (pale blue). The Neu5Ac_b_-[α-2,8]-Neu5Ac_a_-[α-2,3]-Gal_a_ motif is shown in solid sticks, together with the adjacent GalNAc moiety. All superpositionings were carried out in PyMOL [61] using ‘align’ for the protein chains only. Surface, E91 and V296 are from PTA/GT1a. All ‘align’ rmsd values are below 0.16 Å. **B & C** Close view of the van-der-Waals contacts introduced by the E91 side chain present in PTA and LID (C), but not in RA (B). Hydrogen bonds are shown in grey, van-der-Waals contacts in cyan. **D & E** Close view of the hydrophobic contact mediated by V296 in RA and PTA (D), but not by A296 in LID (E). The 4.0 Å hydrophobic contact is not present in the LID strain, whose pocket is opened to the right. Hydrogen bonds are shown in grey, hydrophobic contacts are shown in gold.

Because the differences in tumorigenicity and host spread among strains have been mapped to the glycan binding pocket of VP1, and because GT1a appears to be particularly efficient in facilitating productive infection, we set out to determine how the three strains engage GT1a. By solving the crystal structures of RA and LID VP1 complexed with GT1a using the identical strategy used for the PTA-GT1a complex, we found that the overall binding mode of GT1a is very similar across the three strains (Fig 5A), with a conserved binding mode of the [α-2,8]-linked Neu5Ac_b_. Although the replacement of glutamate with glycine at position 91 leads to a contact area decrease of 33 Å^2^ in RA, the orientation of GT1a in this strain is not altered (compare Fig 5B and 5C). Likewise, the substitution of valine with alanine at position 296 in LID removes a hydrophobic contact but does not affect the conformation of GT1a (Fig 5E; S2 Fig).

The Neu5Ac_a_-Gal_a_-GalNAc linkages in the long arm of GT1a adopt conformations that have been reported in numerous structures (for example [32–34]). While the [α-2,3] linkage between Neu5Ac_a_ and Gal_a_ adopts the conformation that has been reported for DSLNT and 3SL, the branching Neu5Ac_d_-[α-2,3]-Gal_b_ linkage adopts a different conformation, which has been reported for structures containing O-4-substituted galactoses (as in [35,36]). While a higher variability is observed for Neu5Ac-[α-2,8]-Neu5Ac linkages (S2E Fig), this linkage adopts torsion angles that are in agreement with other, related structures such as in the structure of human liver fructose-1,6-bisphosphatase in complex with an allosteric inhibitor [37] or in the complex of tetanus toxin with a GT1b analog [38]. The overall structure is in good agreement with a molecular dynamics simulation using an AMBER force field in an aqueous environment [39]. A well-defined set of water molecules mediates bridged hydrogen bonds between the pyranose moieties, especially between Neu5Ac_b_ and Neu5Ac_d_ (S3 Fig). Due to these steric constraints, the GT1a complexes feature well-defined electron density not only for the binding epitope, but also for the non-binding, branching NeuNAc_d_ in its preferred solution conformation [40], which brings this moiety to about 5 Å near the end of the long arm and gives the glycan the characteristic, horseshoe-like topology that is observable in all complex structures.

### Structures of MuPyV VP1 strains bound to other sialylated glycans

As RA, PTA, and LID VP1 all bind GT1a in a highly similar conformation, we hypothesized that the differences in pathogenicity and spread among the three strains might be due to the recognition of additional carbohydrates by only a subset of MuPyV strains. As shown in Fig 1, the many different gangliosides share a relatively small set of common sialoglycotopes. We therefore investigated the ability of all three VP1 proteins to bind other glycan structures that are representative for these epitopes. We solved structures of VP1 bound to the glycan portions of two of these gangliosides: The GD1a glycan is an established infectious receptor and essentially a truncated version of GT1a lacking the [α-2,8]-linked Neu5Ac_b_ in the long arm. The human milk hexasaccharide DSLNT is the glycan portion of the lacto-series ganglioside 3’-6’-isoLD1 (Fig 1) [41], which is overexpressed in the central nervous system. In contrast to GT1a and GD1a, DSLNT does not contain an [α-2,3]-linked Neu5Ac_d_ as a short arm but instead a branching [α-2,6]-linked Neu5Ac_c_. This structure is similar to a very common epitope on O-linked glycoproteins [42–44]. DSLNT was used in previous studies of MuPyV as a model “pseudoreceptor” [12] and was investigated here to help rationalize these earlier data, to facilitate a comparison among strains, and to establish a binding profile for glycans containing an [α-2,6]-linked sialic acid.

#### GD1a

**Fig 6:**
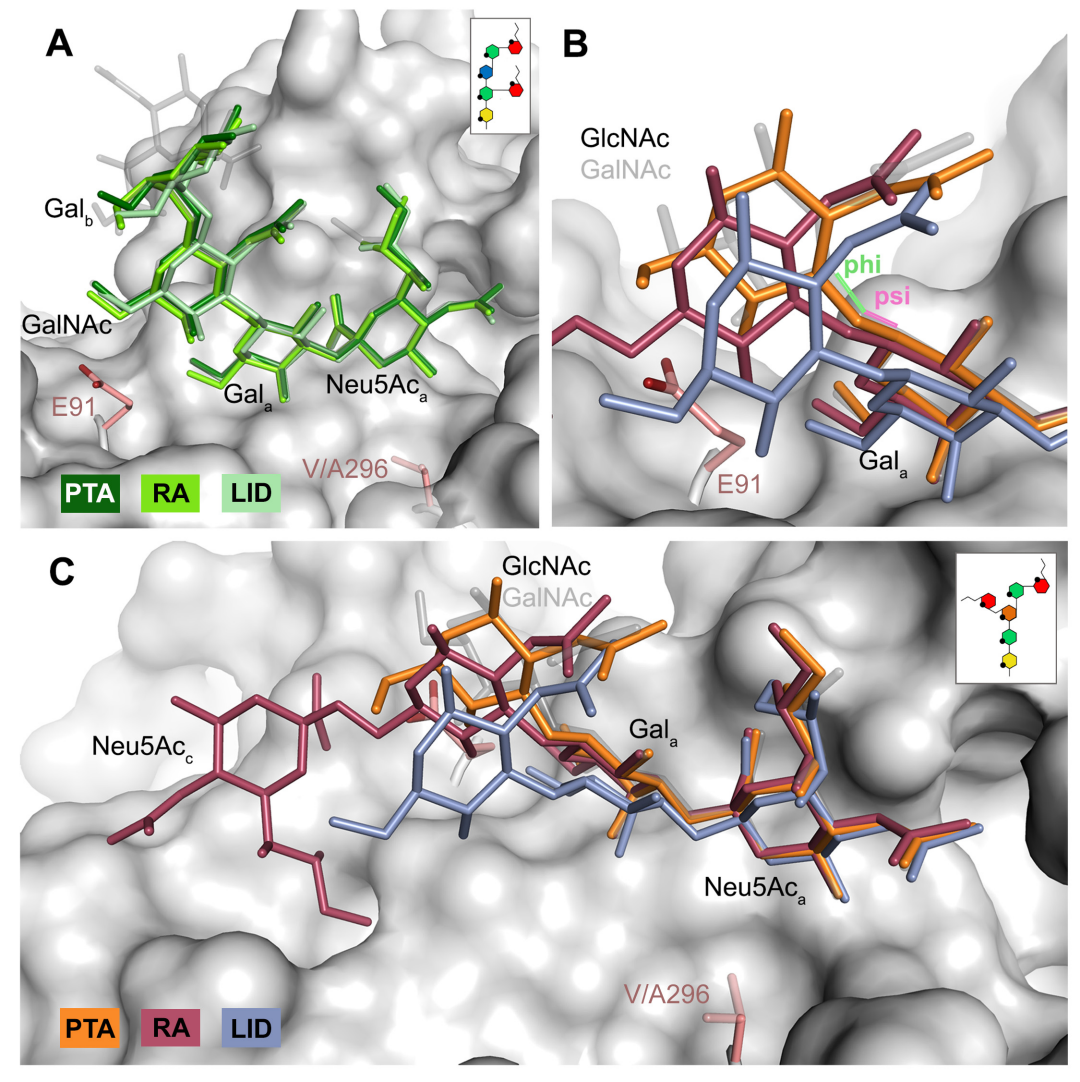
Differences in receptor binding patterns across glycans. **A** Superposition of the binding modes of GD1a to RA (light green), PTA (dark green), and LID (pale green). The sequence of GD1a is shown in the inset. The Neu5Ac_a_-[α-2,3]-Gal_a_ motif is shown in solid sticks, together with the adjacent GalNAc moiety. In all figures, GT1a bound to PTA is overlaid as a grey ghost for comparison, with Neu5Ac_b_ omitted for clarity. Deviations exceeding the atomic error of the structure and alignment rmsd values are only found in the stem region of the sugar, starting at Gal_b_. All superpositionings were carried out in PyMOL [61] using ‘align’ for the protein chains only. Surface, E91 and V296 are from PTA/GT1a. All ‘align’ rmsd values are below 0.16 Å. **B & C** Comparison of the DSLNT binding modes to RA (red), PTA (orange), and LID (violet). In PTA-DSLNT, [α-2,6]-branching causes a 15–20° psi angle shift of the GlcNAc moiety compared to GD1a and GT1a, resulting in a 1 Å sideward twist movement of the stem. In RA-DSLNT, combination of this shift with a 15° shift in the phi angle results in a downward movement of GlcNAc and its branching Neu5Ac_c_ compared to PTA-DSLNT. In LID, the shift is already observable for Gal_a_ and results in the loss of ordered density for GlcNAc. All angles were analyzed with CARP. The sequence of DSLNT is shown in the inset of panel C.

The previously identified MuPyV receptor GD1a is similar to a truncated GT1a structure, containing only a Neu5Ac_a_-[α-2,3]-Gal_a_ motif instead of Neu5Ac_b_-[α-2,8]-Neu5Ac_a_-[α-2,3]-Gal. The disaccharide engages all three strains in a very similar manner (Fig 6A). Neither the longer E91 side chain (in PTA and LID) nor the shorter A296 side chain (in LID) result in an altered conformation of the ligand.

#### DSLNT

The DSLNT glycan terminates in a Neu5Ac_a_-[α-2,3]-Gal_a_ motif, which is the part of the molecule best defined by electron density in all complexes. DSLNT also contains an additional [α-2,6]-linked, branched Neu5Ac_c_ residue, which is not present in either GT1a or GD1a. There is weak electron density for Neu5Ac_c_ in one of the five binding pockets of the RA strain, but it only engages in few interactions [12]. While PTA and LID do bind DSLNT, the complex structures do not show any electron density for the Neu5Ac_c_, indicating that this sugar is conformationally flexible and does not contribute contacts. When bound to the PTA strain, the stem of DSLNT is moderately rearranged (Fig 6B). In comparison to GalNAc in GT1a, the GlcNAc moiety is slightly tilted away from E91 due to a ~20° rotation of the psi angle in the Gal_a_-[β-1,3]-GlcNAc linkage (Fig 6B and 6C, assessed using CARP) that propagates throughout the sugar. In addition, there is no visible electron density for the GlcNAc O6 that is engaged in the [α-2,6]-branching as well as an increased B-Factor variance within the glycan (S4 Fig). The reason for the sideward twist and the missing electron density for Neu5Ac_c_ observed in PTA is probably the electrostatic repulsion between the carboxyl groups of Neu5Ac_c_ and E91. While the charge of E91 is compensated by K186 (Fig 3), as was hypothesized before [12], the two carboxylate groups would come within about 2 Å of one another if DSLNT bound to PTA in the same way as observed in RA. This hypothesis is confirmed by a PTA E91Q mutant that rescued binding of Neu5Ac_c_ (S5 Fig). In turn, when bound to RA, DSLNT exhibits a stronger conformational rearrangement (Fig 6B and 6C). Due to the missing side chain at position 91, the psi angle rotation between Gal_a_ and GlcNAc is accompanied by an additional 15° rotation of the phi angle, bringing the GlcNAc moiety and the attached Neu5Ac_c_ closer to the protein surface [12]. In LID, the valine to alanine mutation at position 296 reduces its van-der-Waals radius. This change results in a broader binding pocket compared to the other strains and the loss of a hydrophobic interaction between position 296 in VP1 and C3 of Neu5Ac_a_ for all glycans. This gives room for a stark alteration in the binding mode of DSLNT that starts with a slight tilt of Neu5Ac_a_ and propagates through the sugar (Fig 6C), ultimately resulting in a prominent sideward shift of the whole glycan stem. The resulting increased conformational freedom of DSLNT is reflected by a lack of electron density in its stem region as well as by an elevated temperature factor variance (S4 Fig). This alteration of the binding mode in LID is likely to be observed for other glycans that are not conformationally restrained by the [α-2,3]-linked Neu5Ac_d_.

### Relative affinities of MuPyV strains for sialylated glycans

Since all three MuPyV strains are able to engage the three different glycan structures in a largely identical manner, we reasoned that the differences in pathogenicity and spread might be attributable to subtle differences in affinity, rather than specificity, among the strains. The affinities of RA VP1 for 3’-sialyllactose and DSLNT were estimated to be in the low mM range [11]. Coupled with the high costs of glycans and the high amount required due to their low binding affinity, weak binding poses technical challenges for classical affinity measurements. We therefore utilized a crystallographic approach to quantitatively compare ligand binding. We crystallized all three VP1 pentamers in the same condition, and soaked each with the oligosaccharide portions of GT1a, GD1a, and DSLNT at different concentrations in parallel. X-ray data of all crystals were collected in the same manner, and the data sets were processed using the same protocol and integrated as described previously [45]. All data sets were processed in the same unit cell, scaled, and the bias-reduced difference electron density around the central Neu5Ac_a_-[α-2,3]-Gal_a_ motif was quantified for each data set (see the Methods section for details). Our crystallization condition contains a high amount of ammonium sulfate, which competes with the carboxyl group of Neu5Ac_a_ and has to be displaced by the carbohydrates. Therefore, our observed binding is weaker than in a physiological setting. However, while not yielding dissociation constants in the traditional sense, this method enables us to compare relative levels of binding across our three different strains and three different glycans.

**Fig 7:**
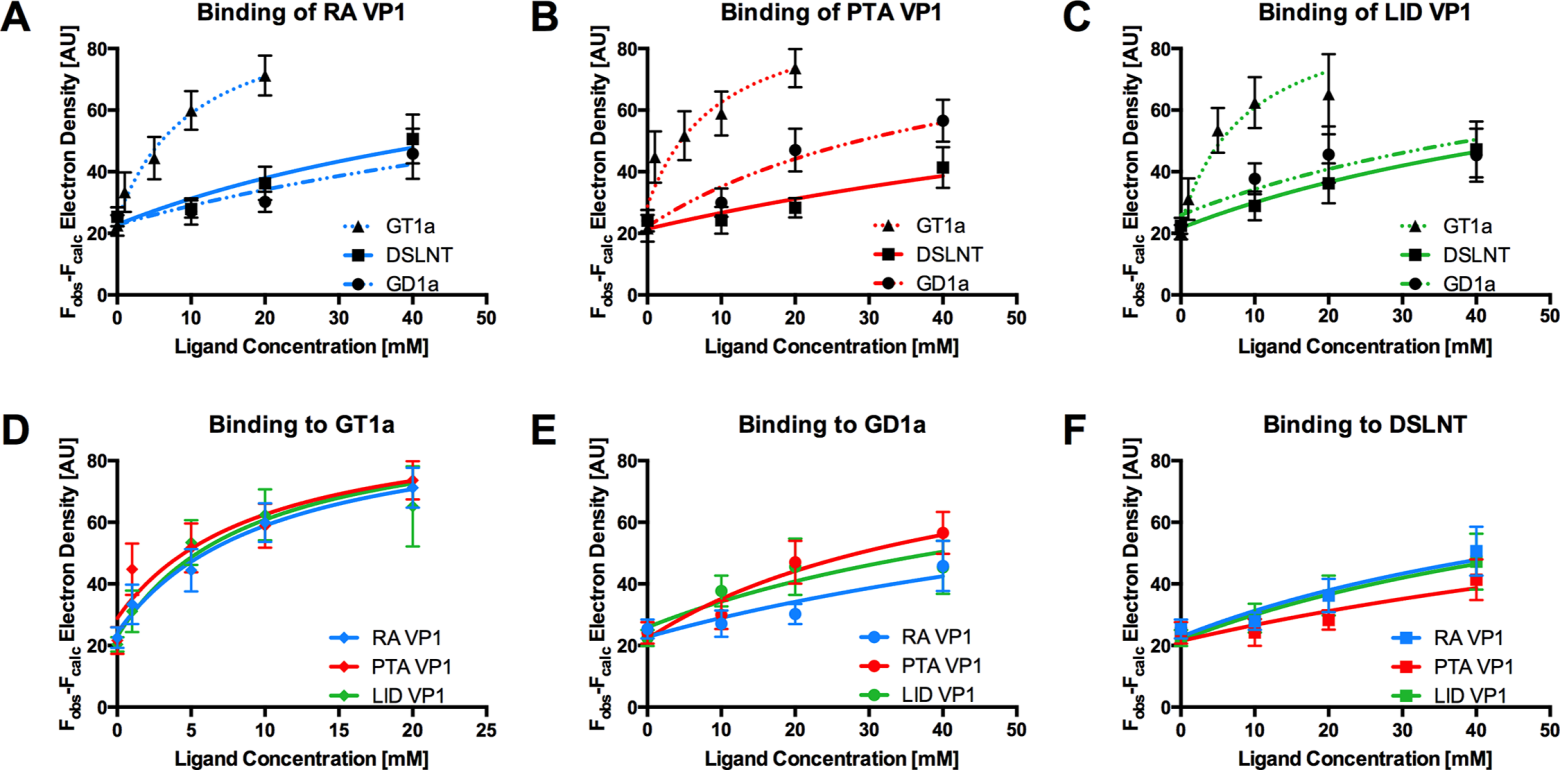
Binding of ligands to MuPyV VP1. The average simulated annealing F_obs_-F_calc_ electron density for the Neu5Ac_a_-[α-2,3]-Gal_a_ in GT1a, GD1a, and DSLNT is plotted against ligand concentration. RA VP1 is colored blue, PTA VP1, is colored red, and LID VP1 is colored green. GT1a is displayed in a dotted line with triangles, GD1a in a dashed line with circles, and DSLNT in a solid line with squares. The error bars correspond to the standard deviation of the mean electron density observed in the five chains of VP1. **A** Electron density of GT1a, GD1a, and DSLNT in RA VP1. **B** Same as in **A**, but for PTA VP1. **C** Same as in **A**, but for LID VP1. **D** Comparison of GT1a-derived electron density in RA VP1, PTA VP1, and LID VP1. **E** Same as in **D**, but for GD1a. **F** Same as in **D**, but for DSLNT.

The GT1a glycan exhibits the strongest binding in all three VP1 variants compared with DSLNT or GD1a (Fig 7A–7C), with no detectable difference between the strains (Fig 7D). This finding is in accord with our ganglioside add-back experiments in cell culture (Fig 2), which consistently showed higher levels of infection mediated by GT1a compared to GD1a. The stronger overall binding of GT1a can be attributed to the additional [α-2,8]-linked sialic acid present in GT1a (Neu5Ac_b_), which contributes several interactions and an increased buried surface area. These contacts seem to outweigh the differences in van der Waals contacts with the side chains of E91 or V296, at least to the extent discernable in our assay.

GD1a binds less well to all strains compared to GT1a. In addition, there are differences in binding strength among the three strains. PTA and LID VP1 appear to bind GD1a at the same level and better compared with RA (Fig 7A–7C and 7E) because these two strains gain additional interaction surface and van-der-Waals contacts from their E91 side chain. This effect is more pronounced than in GT1a, because in GD1a it cannot be masked by the additional contacts of the [α-2,8]-linked Neu5Ac_b_.

DSLNT displays the lowest overall affinity to all strains, with levels comparable to GD1a in RA for all three strains (Fig 7A–7C) despite the DSLNT conformation being slightly different in each VP1 complex (Fig 7F). Neither the blocking of Neu5Ac_c_ binding by E91, nor the increased conformational freedom in LID appears to alter binding affinity. It is possible that Neu5Ac_c_ in RA adopts a conformation that might not be favorable and therefore not heavily contribute to affinity, in spite of the added contact surface. Combined with the fact that electron density for Neu5Ac_c_ could only be observed in one binding pocket of RA VP1 [12], we believe that this conformation is possible but not probable in solution. Instead, an increased number of conformational options might make up for a loss of binding contacts.

## Discussion

Many viruses engage cell-surface glycans to mount an infection, and subtle differences in the recognition of such receptors can be linked with altered tropism and pathogenicity. Examples include the canine parvovirus and feline panleukopenia virus [46,47], the human BK polyomavirus [48], B-lymphotropic polyomavirus [49,50] as well as avian and human influenzaviruses [51,52]. However, MuPyV is a rare example of a virus in which drastic differences in pathogenicity directly correlate with single amino acid substitutions in the viral capsid.

In order to provide a structural basis for understanding the profoundly different pathogenicities of the three MuPyV strains RA, PTA and LID, we have solved structures of their VP1 proteins and characterized their receptor-binding properties. We show that the ganglioside GT1a serves as a MuPyV receptor and promotes infection with higher potency than the previously identified receptors GD1a and GT1b. Structurally, the increased potency of GT1a can be directly explained by a set of additional contacts involving the [α-2,8]-linked Neu5Ac_b_ that is only present in this glycan and that gives it a characteristic horseshoe-like shape. It had previously been suggested that the G91E mutation present in PTA and LID abolishes binding to branched glycans containing [α-2,6]-linked Neu5Ac and thus allows the virus to spread more efficiently in the host [8,11]. However, our analyses show that the presence of a glutamate at position 91 still allows binding of the branched oligosaccharides GT1a, GD1a, or DSLNT to all three strains, albeit with subtle differences in binding affinity. While all three strains bind GT1a with comparable affinity, PTA and LID bind GD1a better than RA. The DSLNT glycan binds similarly to all three strains, with the lowest overall affinity. This is again in line with the structures, which show that the branched Neu5Ac_c_ of DSLNT does not engage in any specific contacts. The limited contacts between Neu5Ac_c_ and RA observed in an earlier structure [12] have to be considered a crystallization artifact as they were only observed in one out of five binding sites, and this visible Neu5Ac_c_ moiety was located near a crystal contact.

The ligand binding promiscuity of MuPyV is surprisingly high. Binding mostly requires a ubiquitous minimal Neu5Ac-[α-2,3]-Gal motif, in agreement with earlier findings [6,7]. It therefore seems plausible that the virus also recognizes other glycans bearing this motif, resulting in differences in pathogenicity and spread. Preliminary studies show that glycans with an N-acetyllactose core (Neu5Ac-[α-2,3]-Gal-[β-1,4]-GlcNAc), as found in neolacto gangliosides such as the predominant ganglioside of peripheral nerve cells, LM1 [53,54], can also be bound in a manner similar to DSLNT and with higher flexibility than GT1a or GD1a (S6 Fig).

Based on our structures, certain requirements that contribute to receptor specificity can be established. For example, branches at Gal-O4 within the minimal motif produce clashes and cannot be tolerated. Therefore, although the GD1a glycan possesses two Neu5Ac-[α-2,3]-Gal motifs, it prefers the one on its longer arm for complex formation. For the same reason, glycans such as GM1 or GM2 that only possess such a branched Neu5Ac-[α-2,3]-Gal epitope cannot engage MuPyV productively. In support of this, the GM1 ganglioside is not able to rescue MuPyV infection of Gang-/- MEFs (Fig 2, [29]), although low-level and probably non-specific interactions with cells can be detected (S1B Fig). GT1b possesses a disialylated arm at Gal_b_ and is monosialylated at Gal_a_. We predict that GT1b engages VP1 with its monosialylated arm. The second, disialylated arm is likely to be accommodated in such a binding mode, and the [α-2,8]-linked sialic acid might contribute additional contacts. Binding via the monosialylated arm is in line with our findings that supplementation of Gang-/- cells with GT1b rescues infection at a level between GD1a and GT1a. Some gangliosides whose glycan epitopes are capable of engaging VP1 *in vitro* might not be infectious receptors *in vivo*, mainly because of steric complications in the context of the cell membrane. For example, while the crystal structure of PTA with the glycan portion of GD3 shows an identical binding mechanism to GT1a (S7 Fig), supplementation of Gang-/- MEFs with GD3 does not restore infectivity [29]. We reason that the glycan stem of GD3 (and of gangliosides with a similar length such as GM3) is too short to allow efficient attachment of the MuPyV capsid to the cell membrane.

The discrepancy in pathogenicity in MuPyV strains that differ only at one single position is stark. In sharp contrast, the differences among receptor binding between the three strains investigated here are subtle, and a correlation of the structural data with the observed pathogenicity profiles remains challenging. One reason for this is that avidity effects in the virus capsid, which can engage many ligands simultaneously, multiply subtle changes in the affinity of capsomers for single glycans. It was shown for influenza viruses that small changes between millimolar binding affinities of single binding sites can result in dramatically altered viral binding properties [52]. As discussed above, we found the main difference between RA and PTA/LID to be a differing affinity for GD1a, which appears to bind better to the latter strains due to the larger E91 side chain. This might facilitate attachment and productive infection by these strains to cells that display GD1a, and may thus give them an advantage over RA. While we could not show differences between the PTA and LID strain in terms of glycan affinity to isolated VP1 pentamers, it is unclear how this translates to avidity effects. As such, it is possible that capsid avidities differ enough to explain the more limited spread of PTA. Although direct correlations cannot be made, it becomes increasingly clear that the virus needs to uphold a delicate equilibrium between efficient infection and release from infected and lysed cells as well as selective affinity for productive receptors. The absence of the RA and LID strains outside the laboratory [26] emphasizes that this equilibrium is affected by minute changes in the receptor binding properties.

The MuPyV receptor pocket can clearly accommodate several related but distinct glycan structures (Figs 1 and 4–6). These structures also decorate glycoproteins on many cell surfaces. It therefore seems likely that MuPyV can also engage glycans that are not attached to gangliosides. For instance, the glycan stem of GD1α, which is very similar to DSLNT and prominent on glycoproteins [42–44], is a likely receptor candidate. The different cell-surface distribution patterns of glycoproteins and gangliosides may likewise influence MuPyV spread [8]. Glycoprotein receptors with unknown identity have in fact been shown to promote non-productive internalization of MuPyV, which in turn elicits innate immune responses by the host [29]. Along these lines, our results suggest that virus particles adhere to and enter ganglioside deficient MEFs to levels that are not significantly lower than for wild-type and ganglioside supplemented Gang-/- cells, although without detectable infection. Although not representative for other cell types, these results suggest that the amount of non-productive “pseudoreceptors” on the MEF cell surface is much higher than anticipated.

Our data demonstrate that varying affinities for different gangliosides are the key determinants of a successful MuPyV infection, in line with earlier reports [6–8]. Perhaps unexpectedly, we also find that (even non-specific) attachment of the virus to a host cell can lead to successful internalization, but that this does not necessarily lead to an infection. Thus, we propose that the ratio between productive (ganglioside bound) and non-productive (ganglioside and glycoprotein bound) glycotopes on the host cell itself or in its microenvironment helps to determine the productivity of infection through diverging entry routes, and that differential affinities to these receptors dictate this equilibrium. The nature of these diverging routes, their underlying driving forces, and potential biological consequences other than immune stimulation [29] remain unknown–as does the point at which they diverge. We cannot exclude the possibility that the distribution and binding properties of (pseudo-)receptors are of importance mostly for the post-entry stage rather than for events taking place at the cell surface. A better understanding of the distribution patterns and densities of glycans on specific cells is clearly needed to fully appreciate the many aspects of pathogenesis and tropism of MuPyV as well as many other glycan-binding viruses.

## Methods

### Ganglioside supplementation and quantification of MuPyV infection

WT and Gang-/- MEFs were seeded onto 96-well Costar 3906 imaging plates in Dulbecco's Modified Eagle's Medium supplemented with 10% fetal bovine serum (FBS). WT (B4+/+St8+/+) and Gang-/- MEFs (B4-/-St8-/-) were provided by Thomas Benjamin at Harvard Medical School. Gangliosides were purchased from Matreya LLC and resuspended in DMSO upon arrival, aliquoted, and stored at -20°C until use. Cells were incubated overnight in serum free media prior to infection. For ganglioside supplemented Gang-/- MEFs, cells were starved in serum free media containing the indicated concentration of ganglioside. Gangliosides were then removed, and cells were washed with serum free media to remove any free ganglioside. Cells were then infected with NG59RA, PTA, and LID MuPyV (MOI ~10–30). At 24 hours post infection cells were washed in phosphate buffered saline and fixed with 4% paraformaldehyde at room temperature for 10 minutes. Cells were then permeabilized with 0.1% Triton X-100, blocked in 10% FBS in PBS, and then stained for the viral protein, T-antigen (E1). Samples were then incubated with Alexa Fluor labeled secondary antibodies (546). Plates were imaged with the Molecular Devices ImageXpress Micro XL High-Content Screener. The percent infected was calculated for each well (5 images were taken per well). Three wells were quantified per sample and the average percent infected, standard error, and standard deviation were calculated for each sample. To quantify infection, T-antigen staining was measured per each DAPI labeled nucleus. For image analysis, the DAPI channel on each image was thresholded, and nuclei were counted using ImageJ (Analyze Particles). These particles were marked as “Regions of Interest” (ROI), and then the average pixel intensity of T-antigen staining was measured for each nucleus (ROI). These were then binned into T-antigen positive or T-antigen negative nuclei to create % infected.

### VP1 immunofluorescence staining

WT and Gang-/- MEFs were seeded onto glass coverslips in Dulbecco's Modified Eagle's Medium supplemented with 10% (FBS). Cells were incubated overnight in serum free media prior to infection. For ganglioside supplementation, Gang-/- MEFs were starved in serum free media containing the indicated concentration of ganglioside. Gangliosides were then removed and cells were washed with serum free media to remove any free ganglioside. Cells were then infected with NG59RA. At indicated times post infection the cells were fixed with 4% paraformaldehyde at room temperature. Cells were blocked in 10% FBS in PBS and then stained for GD1a using mAb MAB5606 (Millipore). Cells were then permeabilized with 0.1% Triton X-100 and stained for the viral proteins, VP1 (I58 antibody) and T-antigen (E1 antibody). Samples were washed and then incubated with Alexa Fluor labeled secondary antibodies (488, 546, 647). Slides were then mounted using DAPI prolong gold mounting media. Slides were imaged with a Nikon A1R confocal microscope. All images were taken as a 9 to 13 step (.25μm) z-stacks on a laser scanning confocal microscope. Each z-stack was aligned and compressed into a max intensity Z projection image.

### Virus binding to ganglioside supplemented Gang-/- MEFs

WT and Gang-/- MEFs were seeded onto a 24 well dish in Dulbecco's Modified Eagle's Medium supplemented with 10% (FBS). Cells were incubated overnight in serum free media prior to infection. For ganglioside supplemented Gang-/- MEFs, cells were starved in serum free media containing the indicated concentration of ganglioside. Gangliosides were then removed and cells were washed with serum free media to remove any free ganglioside. Cells were then infected with either NG59RA, PTA, or LID at an MOI ~10–30 (250 μL/well). At 4 hours post infection 150 μL of virus supernatant was removed and placed into a microcentrifuge tube. This virus supernatant was then used to infect WT MEFs seeded onto a 96-well plate (50 μL/well). The amount of free virus was then quantified as percent of infection of the 96-well reinfection plate. At 24 hours post virus addition the plate was washed in PBS and fixed with 4% PFA at RT for 10 minutes. Cells were then permeabilized with 0.1% Triton X-100, blocked in 10% FBS in PBS, and then stained for the viral protein, T-antigen (E1). Samples were then incubated with Alexa Fluor labeled secondary antibodies (546). Plates were imaged with the Molecular Devices ImageXpress Micro XL High-Content Screener. The percent infected was calculated for each well (5 images were taken per well) as indicated by T-antigen positive nuclei. Three wells were quantified per sample and the average percent infected, standard error, and standard deviation were calculated for each sample. For image analysis, the DAPI channel on each image was thresholded and nuclei were counted using ImageJ (Analyze Particles). These particles were marked as “Regions of Interest” (ROI) and then the average pixel intensity of T-antigen staining was measured for each nucleus (ROI). These were then binned into T-antigen positive or T-antigen negative nuclei to create % infected.

### Expression and purification of VP1 pentamers

DNA encoding residues 33–316 of RA (GenBank # M34958.1) or PTA VP1 (GenBank # PSU27812) was cloned into the expression vector pET15b (Novagen) in frame with an N-terminal hexahistidine tag (His-tag) and a thrombin cleavage site. DNA for LID VP1 (GenBank # PSU27813) was generated by site-directed mutagenesis of PTA VP1 residue 296. VP1 pentamers were overexpressed in *E*. *coli* (BL21) after IPTG induction, and purified by nickel affinity chromatography. The His-tag was removed by thrombin cleavage on column for 72 hours (leaving the non-native residues GSHM at the N-terminus), followed by size exclusion chromatography on a Superdex-200 column.

### Crystallization and crystal soaking

Pure VP1 pentamers were supplemented with 20 mM DTT, concentrated to 7.5–8 mg/mL (RA VP1) or 8.5–9 mg/mL (PTA and LID VP1), and crystallized by sitting-drop vapor diffusion. RA VP1 was crystallized at 20°C against reservoir solutions containing a range of 1.25–1.8 M ammonium sulfate and 1–10% (*v/v*) isopropanol. PTA and LID were crystallized at 4°C against reservoir solutions containing 0.1 M HEPES pH 7–8.5 and 1.6–1.8 M K-Na phosphate. For complex formation, the crystals were soaked in the reservoir solution supplemented with the glycan. The detailed crystallization and soaking procedures are listed in S1 Table. The GT1a and GD1a glycans were purchased from Elicityl SA (France), and the DSLNT glycan was purchased from Carbosynth (United Kingdom).

For concentration-dependent soaking VP1 pentamers of all three strains were crystallized at 20°C against a mother liquor containing 1.5 M ammonium sulfate and 6% (*v/v*) isopropanol. These crystals were soaked in drops of mother liquor containing the appropriate concentration of glycan for 30 minutes.

All crystals were cryoprotected by incubation in mother liquor supplemented with the appropriate concentration of glycan and 25% (*v/v*) glycerol. They were then flash-frozen in liquid nitrogen.

### Structure determination and electron density quantification

Data reduction was carried out in XDS [55], and the structure of native RA VP1 was solved in Molrep [56] using a model generated from the previously solved structure of P16 VP1 (PDB code 1VPN [12]). Other structures were solved by molecular replacement using the RA VP1 structure in Phenix [57]. All structures were completed by alternating rounds of manual model building in Coot [58], followed by restrained coordinate and isomorphous B-factor refinement including TLS refinement and five-fold non-crystallographic symmetry restraints in Refmac5 [59]. TLS parameters were obtained from the TLSMD server [60]. All models agree well with the experimental data and have good geometry (Table 2). The PDB accession codes for the structures are listed in Table 2. Structural figures were prepared in PyMOL [61].

Data collected for concentration-dependent soaking experiments was processed as described above. The unit cell parameters for all datasets were treated as equal for all datasets and isomorphous to the dataset “RA Nat” (S2 Table). They were scaled against “RA Nat” in Scaleit [62] and then subjected to B-factor refinement and simulated annealing in Phenix against models of RA, PTA, or LID VP1, which lacked atoms of all solvent molecules in the receptor binding pocket as well as those of tryptophan residues 98 and 227 as controls. The resulting bias-reduced F_obs_-F_calc_ electron density for Neu5Ac_a_-[α-2,3]-Gal_a_ and the two marker tryptophans was calculated as a summation of values of the grid points in a mask generated 1 Å around these groups using the program Mapman [63]. The overall binding of a sugar at different concentrations influences the electron density of the Neu5Ac_a_-[α-2,3]-Gal_a_ portion, which is included in GT1a, GD1a, and DSLNT. In contrast, it has no effect on the electron density of the marker tryptophan residues, which do not differ significantly for all data points. For each data point, the average density of the five chains was plotted against ligand concentration and submitted to a non-linear least squares fit using the equation

$$Y=\frac{X}{\left(K_{D}+X\right)}\cdot (B_{\max}-B_{0})+B_{0}$$ (1)

where B_max_ was the highest observed electron density value overall (constrained to 95.03 AU) and B_0_ the electron density in the binding pocket at 0 mM ligand concentration. Plotting and fitting was done using the program Prism 6 (GraphPad Software, Inc., La Jolla, California, USA).

## Supporting Information

S1 Fig Gangliosides are required for MuPyV infection, but not for cell surface binding. **(A)** WT, Gang-/- MEFs, and Gang-/- MEFs supplemented with GD1a were infected with NG59RA MuPyV. The MuPyV ganglioside receptor GD1a can be detected on the WT MEFs and GD1a-supplemented Gang-/- MEFs (green), but is absent in Gang-/- MEFs. Virus binds WT, Gang-/-, and GD1a-supplemented Gang-/- MEFs as shown by VP1 staining (red) on the cell surface at 1 hour post infection. At 24 hours post infection, WT and GD1a-supplemented Gang-/- MEFs show robust infection as indicated by nuclear T-antigen staining (magenta). Despite high levels of virus binding, Gang-/- MEFs are completely resistant to infection as shown by lack of T-antigen staining at 24 hours post infection. **(B)** Gang-/- MEFs were supplemented with 2μM GD1a, GT1b, GT1a, GD1b, and GM1 followed by infection with RA, PTA, and LID MuPyV. At 4 hours post infection, virus supernatant was removed and the amount of free virus was quantified for each sample by re-infection of WT MEFs. Virus bound to all cells at similar levels, and there were no significant differences in virus binding to infectious *versus* non-infectious ganglioside receptors. Error bars are standard error, and virus binding to WT MEFs is normalized to one.
(TIF)

S2 Fig CARP Plots of GT1a bound to the PTA VP1 pentamer. The observed phi and psi torsion angles for the linkages occurring in the PTA-GT1a complex have been plotted and compared to other linkages found in the PDB using CARP with the crystallographic definition of torsion angles. The observed linkages are: Neu5Ac-[α-2,3]-Gal (**A,B**), Gal-[β-1,3]-GalNAc (**C**), GalNAc-[β-1,4]-Gal (**D**), and Neu5Ac-[α-2,8]-Neu5Ac (**E**). The inlay on the lower right shows the schematic and observed structure of GT1a. The linkages are named according to the panels; the coloring of the glycan rings was adopted from Fig 1.
(TIF)

S3 Fig Ordered water molecules between the two branches of GT1a. Possible hydrogen bonds between the glycan and ordered water molecules are depicted in grey. In GD1a, the glycerol-like tail of Neu5Ac_a_ could principally also stabilize the glycan, but preferentially adopts a conformation that does not participate in intramolecular contacts.
(TIF)

S4 Fig B-factor variance across different strains and glycans. The glycans are colored by B factor on an absolute scale between 0 (dark blue) and 80 (deep red). GT1a/PTA is shown as a grey ghost for comparison. For GD1a and DSLNT, the intramolecular B-factor variance when bound to LID is considerably higher, while values for GT1a are comparable to the other strains.
(TIF)

S5 Fig Mutation to a glutamine at position 91 of PTA VP1 restores the DSLNT binding mode of RA VP1. Shown are the superimposed DSLNT complex structures of RA (PDB-ID 1VPS [12], transparent red) and PTA E91Q (yellow, r.m.s.d. value for the superposition in PyMOL: 0.159 Å). An F_obs_-F_calc_ omit map (2σ, carved 1.6 Å around the ligand) is shown for the PTA E91Q complex. On the lower right, DSLNT is represented schematically. As for RA VP1, visible electron density for Neu5Ac_c_ in PTA E91Q can be seen in one of the five chains.
(TIF)

S6 Fig The reduced van-der-Waals radius at position 296 in LID allows for a more versatile glycan binding. The van-der-Waals radius of 3.5 Å is indicated as dotted sphere for V296 (PTA, yellow) and A296 (LID, blue). The mutation opens the pocket to one side and allows for a more flexible binding mode of glycans without internal stabilization (DSLNT and 3’-N-Acetyl-sialyllactosamine (3’-SLN), the prototype glycan of the LM1 ganglioside). O4 of Gal_a_ is pointing directly towards the surface in all complexes. In this binding mode, no branching at this point (as is the case e.g. for GD1b) can be tolerated. Glycans that adopt a binding mode similar to the rigid GT1a are colored in green tones, glycans that exhibit shifts of their moieties are colored in shades of red. GT1a bound to PTA is shown as a grey ghost for comparison.
(TIF)

S7 Fig GD3 binding to VP1. The complex structures of PTA VP1 with GT1a (dark blue) and GD3 (light pink) are superimposed in PyMOL. On the upper right, the structure of GD3 is represented schematically.
(TIF)

S1 Table Crystallization and soaking conditions. (DOCX)

S2 Table Data set statistics for concentration-dependent soaking experiments. (DOCX)
