# Supplementary figures and images for: Structural and Functional Analysis of Murine Polyomavirus Capsid Proteins Establish the Determinants of Ligand Recognition and Pathogenicity
Source: PLoS Pathog. 2015 Oct 16;11(10):e1005104. doi: 10.1371/journal.ppat.1005104 (PMC4608799; doi:10.1371/journal.ppat.1005104)

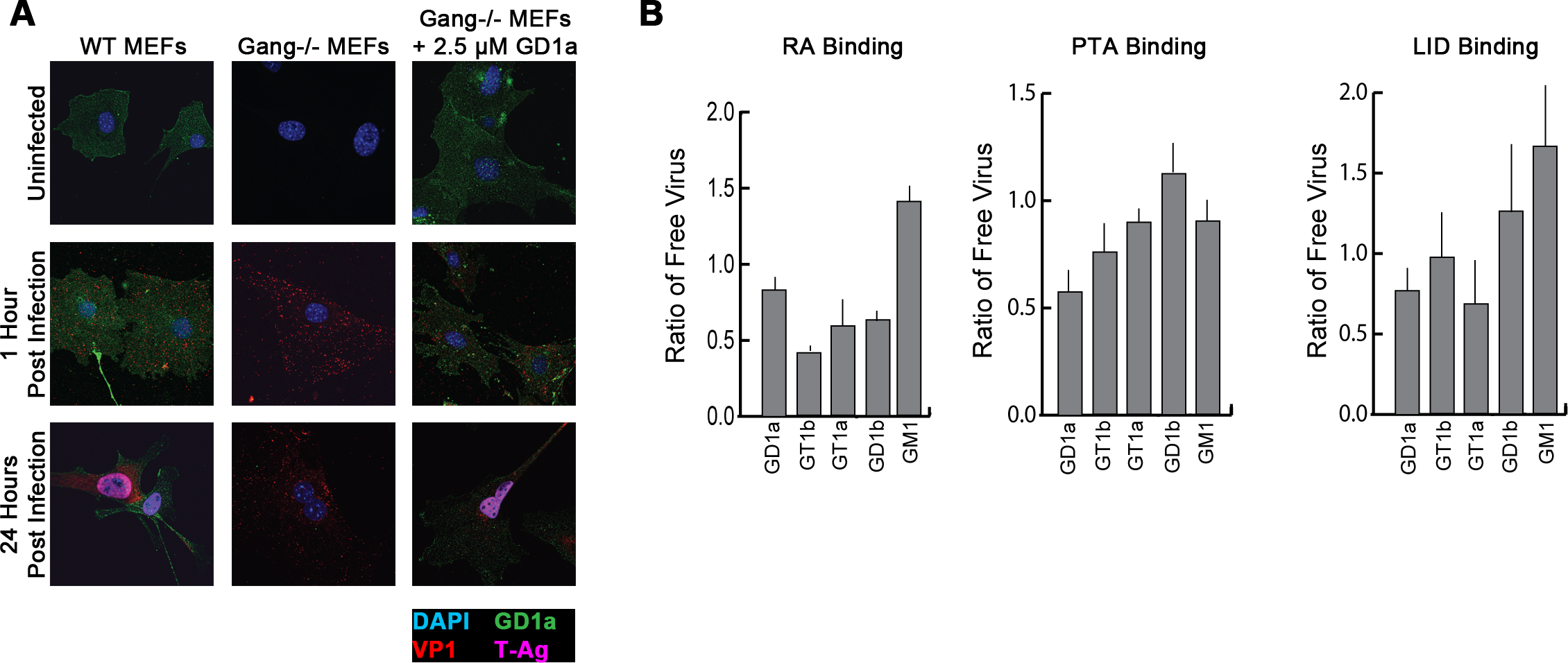

Supplement: S1 Fig — (A) WT, Gang-/- MEFs, and Gang-/- MEFs supplemented with GD1a were infected with NG59RA MuPyV. The MuPyV ganglioside receptor GD1a can be detected on the WT MEFs and GD1a-supplemented Gang-/- MEFs (green), but is absent in Gang-/- MEFs. Virus binds WT, Gang-/-, and GD1a-supplemented Gang-/- MEFs as shown by VP1 staining (red) on the cell surface at 1 hour post infection. At 24 hours post infection, WT and GD1a-supplemented Gang-/- MEFs show robust infection as indicated by nuclear T-antigen staining (magenta). Despite high levels of virus binding, Gang-/- MEFs are completely resistant to infection as shown by lack of T-antigen staining at 24 hours post infection. (B) Gang-/- MEFs were supplemented with 2μM GD1a, GT1b, GT1a, GD1b, and GM1 followed by infection with RA, PTA, and LID MuPyV. At 4 hours post infection, virus supernatant was removed and the amount of free virus was quantified for each sample by re-infection of WT MEFs. Virus bound to all cells at similar levels, and there were no significant differences in virus binding to infectious versus non-infectious ganglioside receptors. Error bars are standard error, and virus binding to WT MEFs is normalized to one. (TIF) [file ppat.1005104.s001.tif]

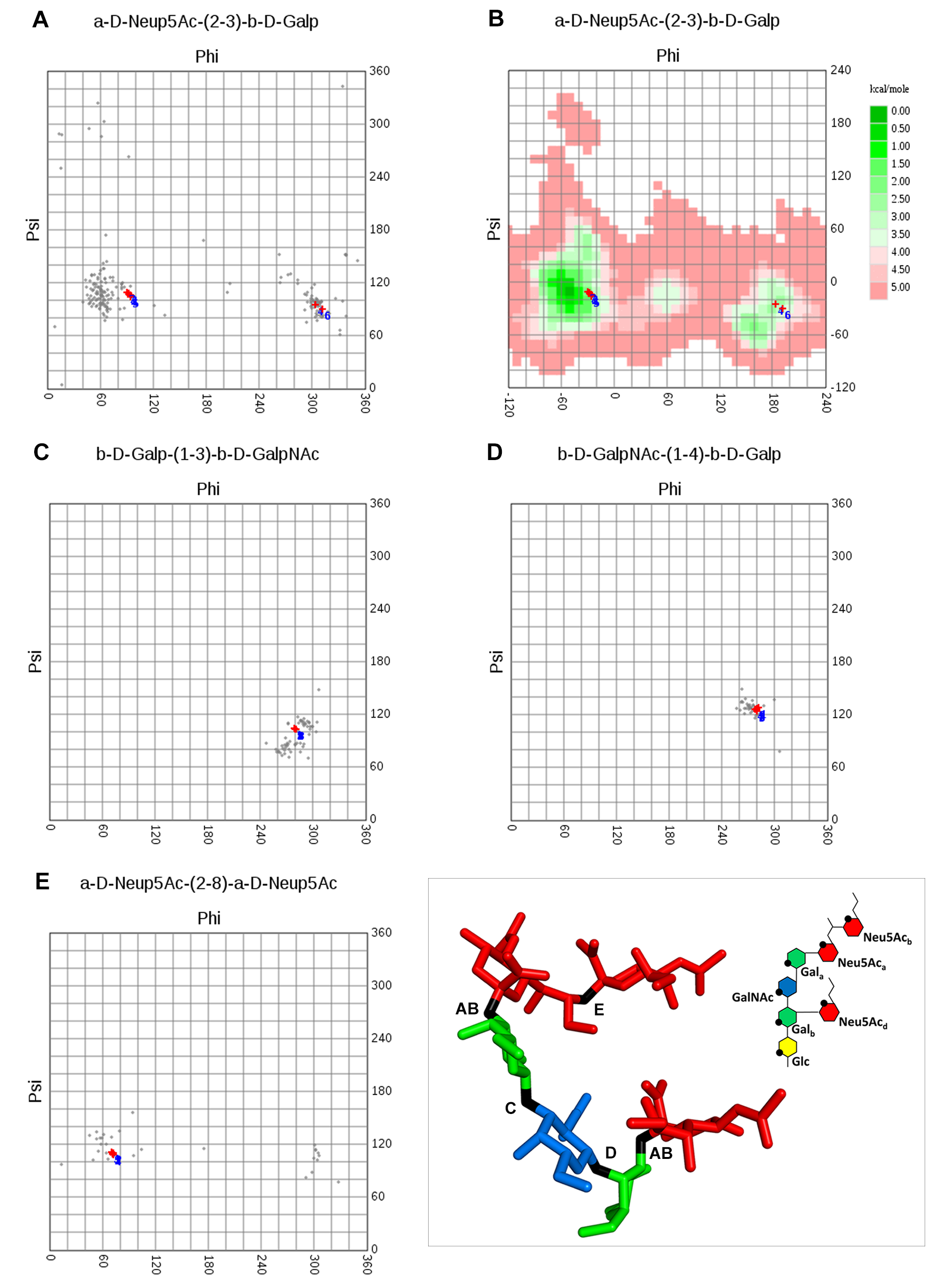

Supplement: S2 Fig — The observed phi and psi torsion angles for the linkages occurring in the PTA-GT1a complex have been plotted and compared to other linkages found in the PDB using CARP with the crystallographic definition of torsion angles. The observed linkages are: Neu5Ac-[α-2,3]-Gal (A,B), Gal-[β-1,3]-GalNAc (C), GalNAc-[β-1,4]-Gal (D), and Neu5Ac-[α-2,8]-Neu5Ac (E). The inlay on the lower right shows the schematic and observed structure of GT1a. The linkages are named according to the panels; the coloring of the glycan rings was adopted from Fig 1. (TIF) [file ppat.1005104.s002.tif]

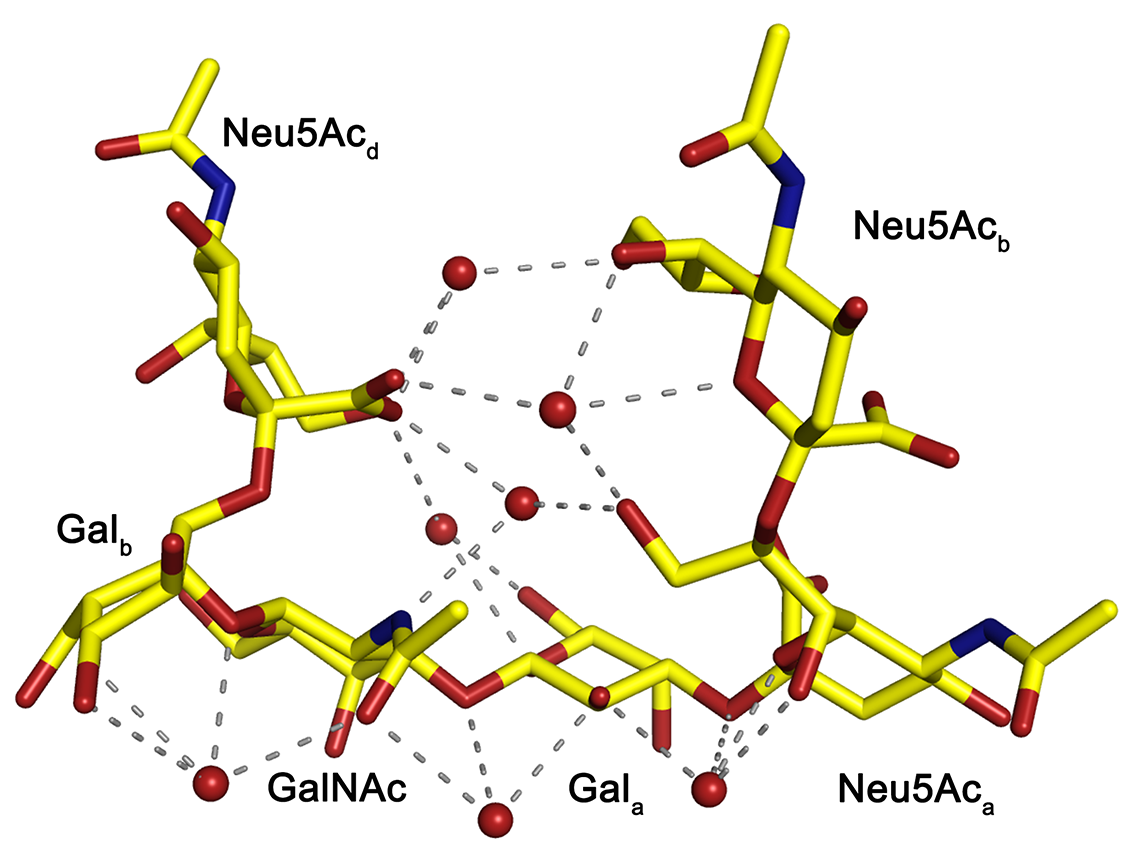

Supplement: S3 Fig — Possible hydrogen bonds between the glycan and ordered water molecules are depicted in grey. In GD1a, the glycerol-like tail of Neu5Aca could principally also stabilize the glycan, but preferentially adopts a conformation that does not participate in intramolecular contacts. (TIF) [file ppat.1005104.s003.tif]

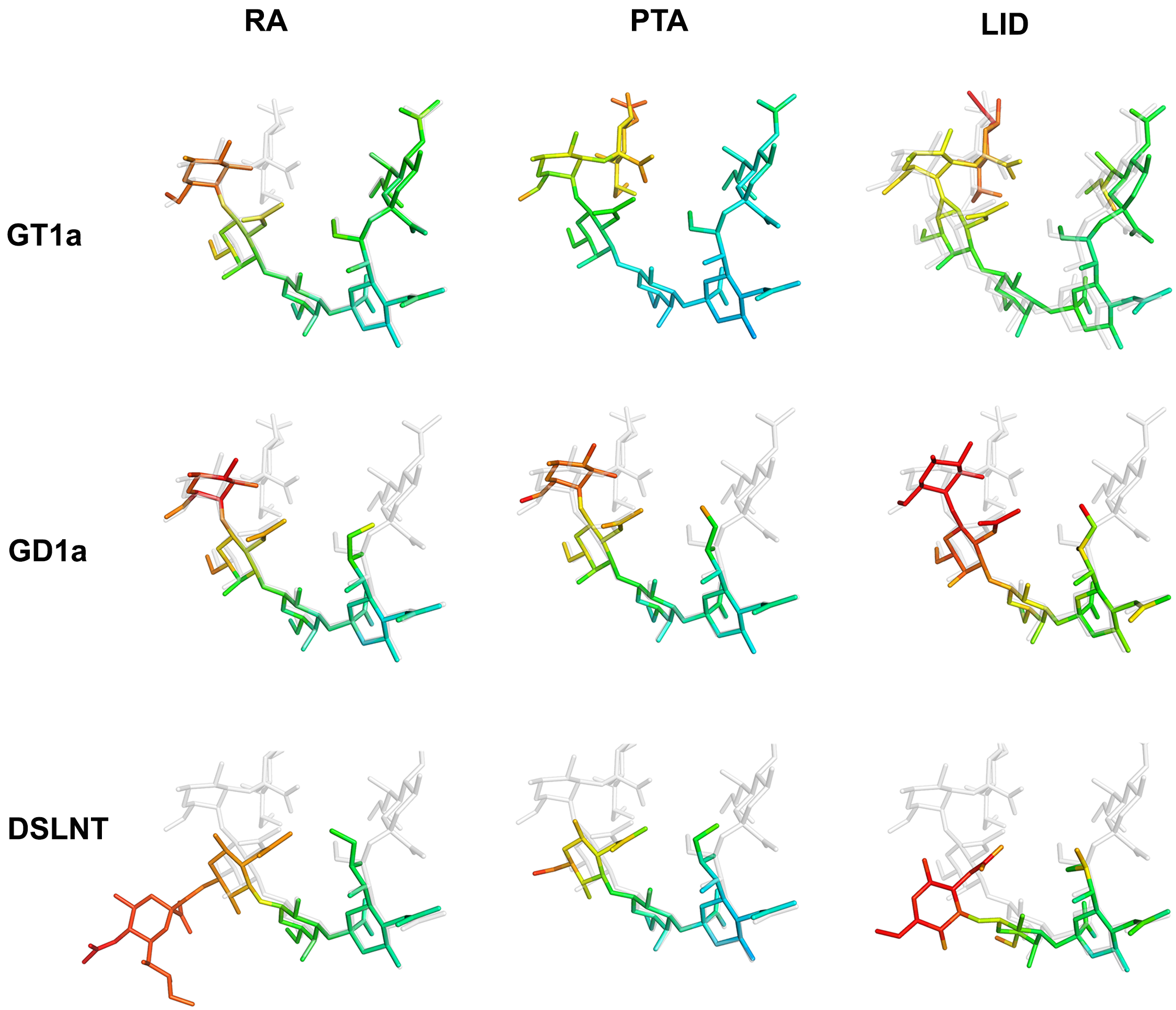

Supplement: S4 Fig — The glycans are colored by B factor on an absolute scale between 0 (dark blue) and 80 (deep red). GT1a/PTA is shown as a grey ghost for comparison. For GD1a and DSLNT, the intramolecular B-factor variance when bound to LID is considerably higher, while values for GT1a are comparable to the other strains. (TIF) [file ppat.1005104.s004.tif]

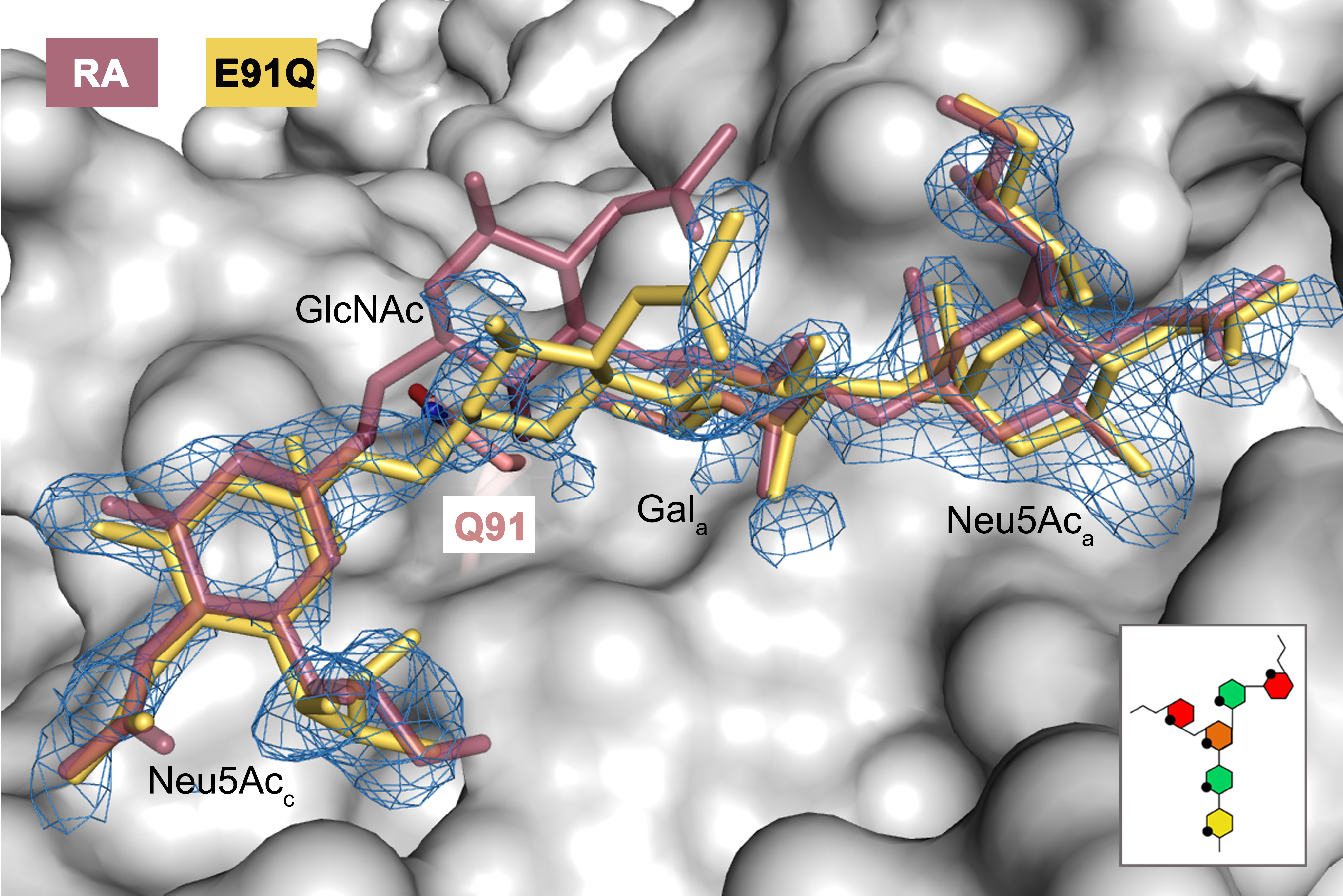

Supplement: S5 Fig — Shown are the superimposed DSLNT complex structures of RA (PDB-ID 1VPS [12], transparent red) and PTA E91Q (yellow, r.m.s.d. value for the superposition in PyMOL: 0.159 Å). An Fobs-Fcalc omit map (2σ, carved 1.6 Å around the ligand) is shown for the PTA E91Q complex. On the lower right, DSLNT is represented schematically. As for RA VP1, visible electron density for Neu5Acc in PTA E91Q can be seen in one of the five chains. (TIF) [file ppat.1005104.s005.tif]

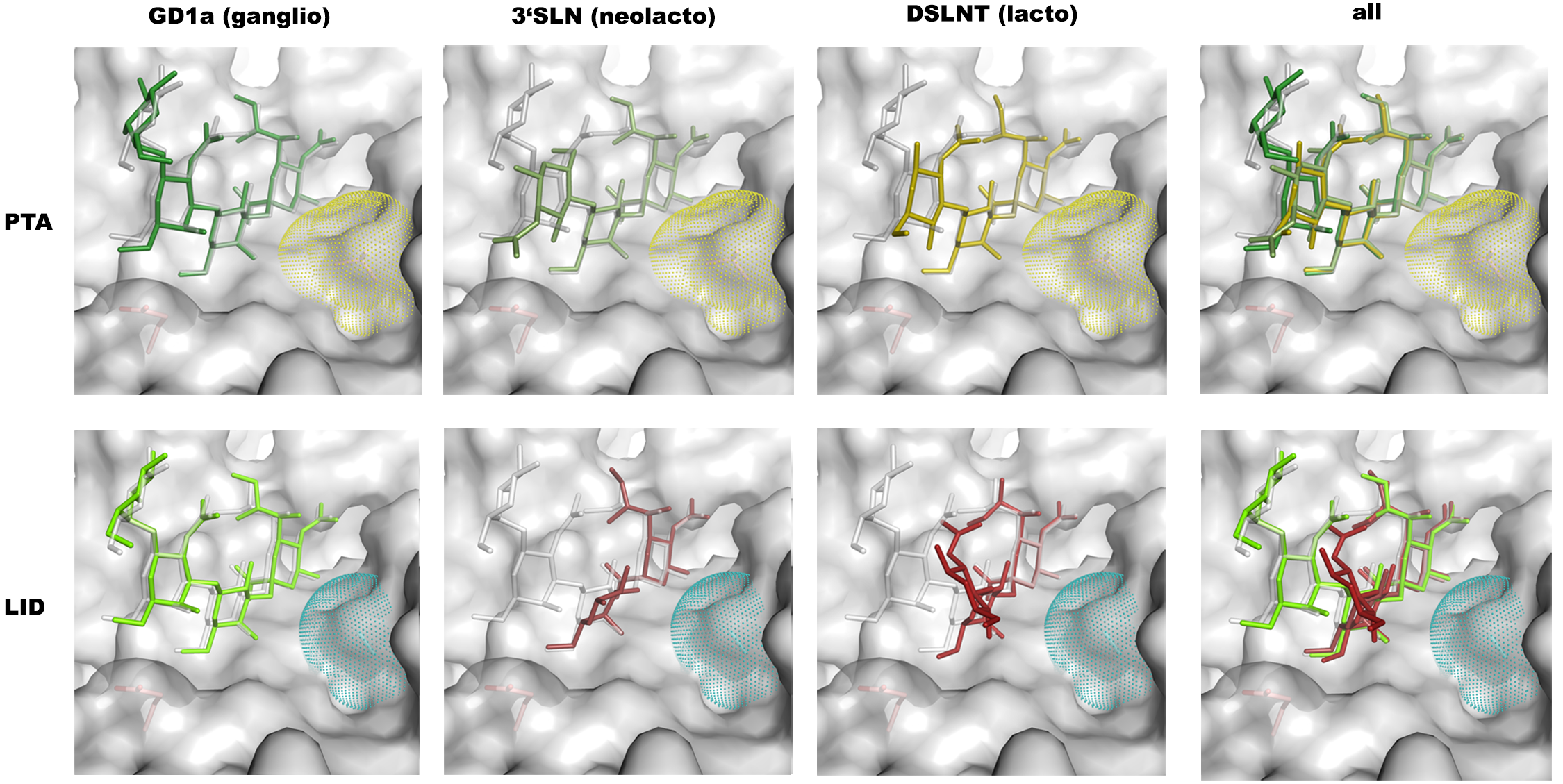

Supplement: S6 Fig — The van-der-Waals radius of 3.5 Å is indicated as dotted sphere for V296 (PTA, yellow) and A296 (LID, blue). The mutation opens the pocket to one side and allows for a more flexible binding mode of glycans without internal stabilization (DSLNT and 3’-N-Acetyl-sialyllactosamine (3’-SLN), the prototype glycan of the LM1 ganglioside). O4 of Gala is pointing directly towards the surface in all complexes. In this binding mode, no branching at this point (as is the case e.g. for GD1b) can be tolerated. Glycans that adopt a binding mode similar to the rigid GT1a are colored in green tones, glycans that exhibit shifts of their moieties are colored in shades of red. GT1a bound to PTA is shown as a grey ghost for comparison. (TIF) [file ppat.1005104.s006.tif]

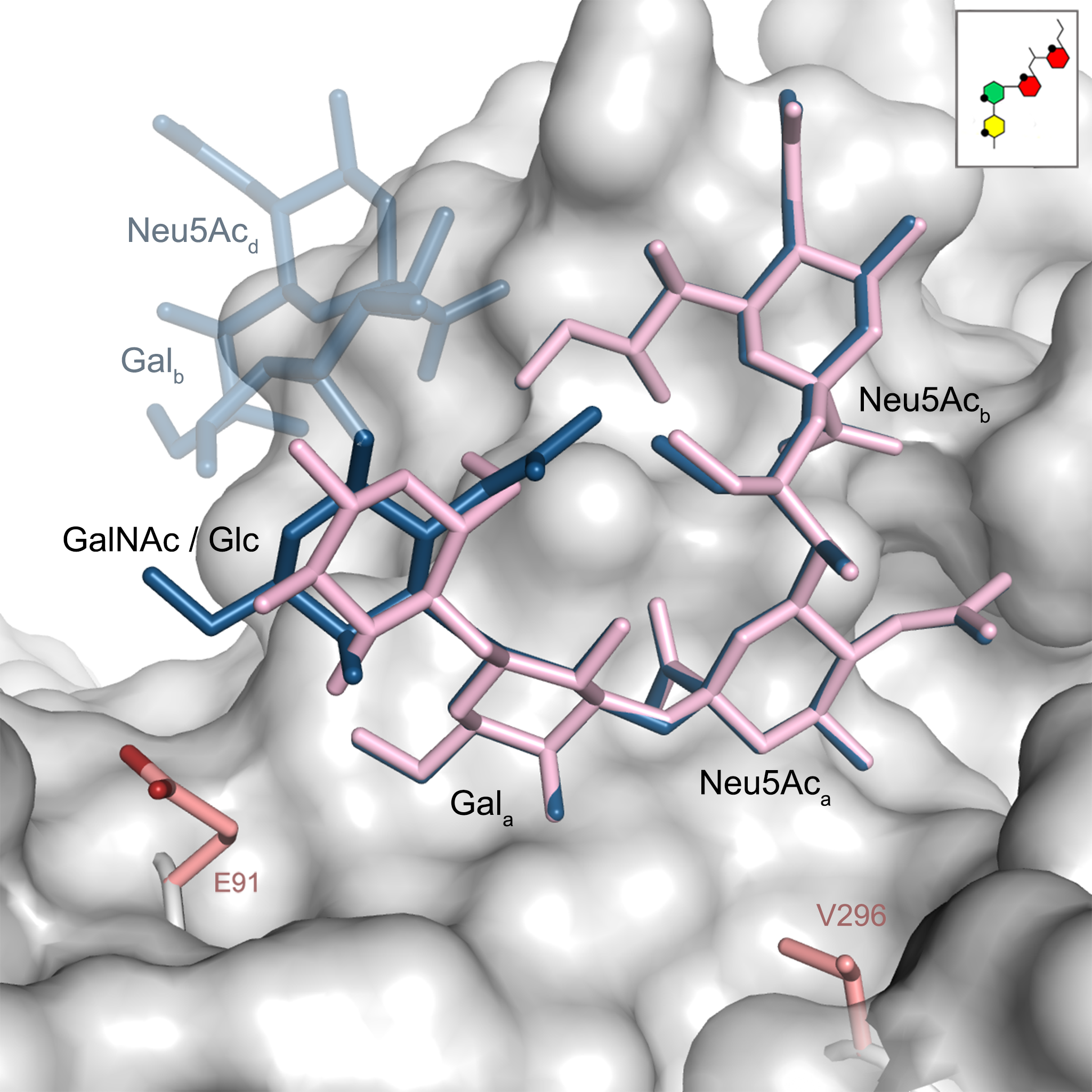

Supplement: S7 Fig — The complex structures of PTA VP1 with GT1a (dark blue) and GD3 (light pink) are superimposed in PyMOL. On the upper right, the structure of GD3 is represented schematically. (TIF) [file ppat.1005104.s007.tif]
