# Supplementary material for: Structural and Functional Analysis of Murine Polyomavirus Capsid Proteins Establish the Determinants of Ligand Recognition and Pathogenicity
Source: PLoS Pathog. 2015 Oct 16;11(10):e1005104. doi: 10.1371/journal.ppat.1005104 (PMC4608799; doi:10.1371/journal.ppat.1005104)
Supplement: S1 Table — (DOCX) [file ppat.1005104.s008.docx]

|  | RA VP1 + GT1a | RA VP1 + GD1a | PTA VP1 | PTA VP1 + GT1a | PTA VP1 + DSLNT | PTA VP1 + GD1a |
| --- | --- | --- | --- | --- | --- | --- |
| Crystallization condition | 1.7 M ammonium sulfate  4.9% (*v/v*) isopropanol | 1.5 M ammonium sulfate  6% (*v/v*) isopropanol | 0.1 M HEPES pH 7.5  1 M NaH_2_PO_4_  0.8 M K_2_HPO_4_ | 0.1 M HEPES pH 8.0  0.8 M NaH_2_PO_4_  1 M K_2_HPO_4_ | 0.1 M HEPES pH 8.5  1 M NaH_2_PO_4_  0.8 M K_2_HPO_4_ | 0.1 M HEPES pH 7.5  1 M NaH_2_PO_4_  0.8 M K_2_HPO_4_ |
| Soaking procedure | 15 minutes in crystallization condition supplemented with 20 mM GT1a oligosaccharide | 1 hour in crystallization condition supplemented with 100 mM GD1a oligosaccharide | --- | 1 hour in crystallization condition supplemented with 20 mM GT1a oligosaccharide | 1 hour in crystallization condition supplemented with 20 mM DSLNT oligosaccharide | 1 hour in crystallization condition supplemented with 20 mM GT1a oligosaccharide |
