# Supplementary material for: Structural and Functional Analysis of Murine Polyomavirus Capsid Proteins Establish the Determinants of Ligand Recognition and Pathogenicity
Source: PLoS Pathog. 2015 Oct 16;11(10):e1005104. doi: 10.1371/journal.ppat.1005104 (PMC4608799; doi:10.1371/journal.ppat.1005104)
Supplement: S2 Table — (DOCX) [file ppat.1005104.s009.docx]

| **Data set** | | **RA Native** | **RA + 1 mM GT1a** | **RA + 5 mM GT1a** | **RA + 10 mM GT1a** | **RA + 20 mM GT1a** | **PTA Native** | **PTA + 1 mM GT1a** | **PTA + 5 mM GT1a** | **PTA + 10 mM GT1a** |
| --- | --- | --- | --- | --- | --- | --- | --- | --- | --- | --- |
| Beamline | | SLS, X06DA | SLS, X06DA | SLS, X06SA | SLS, X06DA | SLS, X06DA | SLS, X06DA | SLS, X06DA | SLS, X06SA | SLS, X06SA |
| Space Group | | P3_1_21 | | | | | | | | |
| Cell Dimensions | |  | | | | | | | | |
|  | a=b, c [Å] | 218.93, 99.45 | | | | | | | | |
|  | α=β, γ [°] | 90, 120 | | | | | | | | |
| Resolution [Å] | | 35-2.66 (2.73-2.66) | | | | | | | | |
| R_meas_ [%] | | 4.6 (12.6) | 6.2 (16.1) | 8.5 (12.0) | 5.0 (15.9) | 11.0 (13.6) | 6.3 (15.0) | 3.0 (6.7) | 11.8 (24.3) | 11.1 (39.9) |
| I/σ(I) | | 28.4 (11.8) | 22.4 (9.9) | 14.9 (10.2) | 27.0 (9.0) | 13.6 (10.1) | 18.5 (8.8) | 36.9 (18.1) | 11.8 (6.0) | 11.0 (3.2) |
| Completeness [%] | | 99.4 (99.6) | 95.1 (95.8) | 97.3 (98.5) | 98.6 (99.5) | 95.8 (95.7) | 98.0 (99.8) | 94.5 (97.1) | 97.5 (99.0) | 98.0 (98.9) |
| Redundancy | | 3.6 (3.8) | 4.9 (4.7) | 3.8 (3.6) | 3.8 (3.5) | 5.2 (4.9) | 3.6 (3.8) | 3.3 (3.1) | 5.7 (5.8) | 2.7 (2.8) |
|  | | | | | | | | | | |
| **Data set** | | **LID + 1 mM GT1a** | **LID + 5 mM GT1a** | **LID + 10 mM GT1a** | **LID + 20 mM GT1a** | **RA + 10 mM DSLNT** | **RA + 20 mM DSLNT** | **RA + 40 mM DSLNT** | **PTA + 10 mM DSLNT** | **PTA + 20 mM DSLNT** |
| Beamline | | SLS, X06DA | SLS, X06SA | SLS, X06SA | SLS, X06DA | SLS, X06DA | SLS, X06DA | SLS, X06DA | SLS, X06DA | SLS, X06DA |
| Space Group | | P3_1_21 | | | | | | | | |
| Cell Dimensions | |  | | | | | | | | |
|  | a=b, c [Å] | 218.93, 99.45 | | | | | | | | |
|  | α=β, γ [°] | 90, 120 | | | | | | | | |
| Resolution [Å] | | 35-2.66 (2.73-2.66) | | | | | | | | |
| R_meas_ [%] | | 6.5 (21.3) | 7.5 (11.9) | 7.3 (11.5) | 8.1 (30.3) | 7.2 (23.0) | 5.5 (15.7) | 7.0 (18.9) | 15.6 (34.8) | 3.7 (7.3) |
| I/σ(I) | | 23.1 (7.2) | 20.1 (11.9) | 16.5 (10.5) | 20.7 (6.9) | 14.4 (4.9) | 17.8 (6.8) | 20.5 (9.2) | 7.9 (3.6) | 31.6 (17.0) |
| Completeness [%] | | 96.7 (98.4) | 99.0 (98.7) | 96.7 (98.0) | 96.8 (97.8) | 94.4 (97.8) | 96.2 (91.5) | 98.6 (99.6) | 93.6 (96.0) | 97.6 (97.4) |
| Redundancy | | 4.8 (4.4) | 5.6 (5.4) | 3.7 (3.5) | 6.1 (6.1) | 2.7 (2.3) | 2.3 (2.2) | 5.2 (5.3) | 4.3 (4.1) | 3.8 (3.5) |

| **Data set** | | **LID + 10 mM DSLNT** | **LID + 20 mM DSLNT** | **LID + 40 mM DSLNT** | **RA + 10 mM GD1a** | **RA + 20 mM GD1a** | **RA + 40 mM GD1a** | **PTA + 10 mM GD1a** | **PTA + 20 mM GD1a** | **PTA + 40 mM GD1a** |
| --- | --- | --- | --- | --- | --- | --- | --- | --- | --- | --- |
| Beamline | | SLS, X06SA | SLS, X06SA | SLS, X06SA | SLS, X06DA | SLS, X06SA | SLS, X06SA | SLS, X06DA | SLS, X06SA | SLS, X06SA |
| Space Group | | P3_1_21 | | | | | | | | |
| Cell Dimensions | |  | | | | | | | | |
|  | a=b, c [Å] | 218.93, 99.45 | | | | | | | | |
|  | α=β, γ [°] | 90, 120 | | | | | | | | |
| Resolution [Å] | | 35-2.66 (2.73-2.66) | | | | | | | | |
| R_meas_ [%] | | 10.2 (15.8) | 10.6 (24.6) | 7.8 (13.4) | 6.9 (25.3) | 12.7 (27.8) | 3.4 (6.7) | 12.9 (32.6) | 11.3 (15.2) | 9.1 (15.2) |
| I/σ(I) | | 14.3 (9.3) | 12.3 (5.9) | 16.8 (9.8) | 15.7 (4.5) | 8.0 (3.8) | 42.5 (25.1) | 10.7 (4.9) | 12.1 (8.2) | 13.8 (8.9) |
| Completeness [%] | | 93.3 (95.9) | 97.8 (99.4) | 95.8 (96.7) | 97.0 (92.5) | 94.8 (94.4) | 98.9 (98.5) | 92.8 (95.3) | 97.4 (99.1) | 99.6 (99.9) |
| Redundancy | | 5.9 (5.6) | 4.8 (4.6) | 4.4 (4.2) | 2.3 (2.2) | 2.7 (2.5) | 5.8 (6.0) | 5.2 (4.7) | 4.3 (4.0) | 4.2 (4.5) |
|  | | | | | | | | | | |
| **Data set** | | **LID + 20 mM GD1a** | **LID + 20 mM DSLNT** |  |  |  |  |  |  |  |
| Beamline | | SLS, X06DA | PETRA-III, P-13 |  |  |  |  |  |  |  |
| Space Group | | P3_1_21 | |  |  |  |  |  |  |  |
| Cell Dimensions | |  | |  |  |  |  |  |  |  |
|  | a=b, c [Å] | 218.93, 99.45 | |  |  |  |  |  |  |  |
|  | α=β, γ [°] | 90, 120 | |  |  |  |  |  |  |  |
| Resolution [Å] | | 35-2.66 (2.73-2.66) | |  |  |  |  |  |  |  |
| R_meas_ [%] | | 12.2 (28.6) | 5.6 (23.0) |  |  |  |  |  |  |  |
| I/σ(I) | | 9.6 (4.9) | 20.4 (5.7) |  |  |  |  |  |  |  |
| Completeness [%] | | 97.1 (98.8) | 96.5 (98.1) |  |  |  |  |  |  |  |
| Redundancy | | 3.7 (3.8) | 2.9 (3.0) |  |  |  |  |  |  |  |
